# Integrated anatomical and functional connectivity mapping in episodic migraine: a spectral graph theory approach

**DOI:** 10.1038/s41598-026-53726-7

**Published:** 2026-05-20

**Authors:** Gonçalo Grácio, Ana Matoso, Inês Esteves, Ana R. Fouto, Amparo Ruiz-Tagle, Gina Caetano, Raquel Gil-Gouveia, Patrícia Figueiredo, Rita G. Nunes, Sérgio Pequito

**Affiliations:** 1 Institute for Systems and Robotics – Lisboa and Department of Electrical and Computer Engineering, Instituto Superior Técnico, University of Lisbon, Lisbon, Portugal; 2 Institute for Systems and Robotics - Lisboa and Department of Bioengineering, Instituto Superior Técnico, University of Lisbon, Lisbon, Portugal; 3 Neurology Department, Hospital da Luz, Lisbon, Portugal; 4 Center for Interdisciplinary Research in Health, Universidade Católica Portuguesa, Lisbon, Portugal; 5 Algarve Biomedical Center, Faro, Portugal

**Keywords:** Neurology, Neuroscience

## Abstract

Migraine disproportionately affects women, yet how migraine physiology reshapes large-scale brain communication remains unclear. We combined diffusion-weighted imaging (DWI) and resting-state fMRI in female participants (14 patients with episodic migraine without aura; 15 matched healthy controls) to test how direct and indirect anatomical communication paths in the brain can predict brain function. We used a spectral mapping framework that isolates the contribution of communication paths of a specific length and evaluated how well brain structure predicts brain function within individuals. Analyses of individual path lengths revealed a non-monotonic dissociation: no difference at one-step (direct) paths, but higher mapping accuracy in patients at intermediate indirect scales (four and five steps). At longer scales, contributions attenuated in both groups. Spatial correspondence analyses localized patient-specific effects to default mode network subsystems across multiple atlases. These findings indicate that migraine-related dysfunction reflects altered mesoscale structure-function integration along indirect anatomical routes, and they provide a general approach to dissect structure-function coupling by communication scale in disease.

## Introduction

Migraine is a highly prevalent and disabling neurological disorder that affects roughly 1.3 billion people worldwide, ranking as the *second* most common disease in the global burden-of-disease hierarchy^1^. Its impact is especially pronounced in women, who are almost three times more likely than men to experience migraine, and in individuals younger than 50 years, for whom migraine is the leading cause of disability^2^.

Clinically, migraine is divided into two subtypes: episodic migraine, defined as fewer than 15 headache days per month, and chronic migraine, defined as 15 or more headache days per month^3^. Beyond the pain itself, migraine is linked to a broad spectrum of comorbidities that exacerbate individual suffering and generate substantial socioeconomic costs^4^.

Approximately 20% of women with migraine report attacks in at least two-thirds of their menstrual cycles^5^, and these menstrual attacks tend to be more severe and less responsive to treatment than non-menstrual attacks^5^. Hormonal changes during the menstrual cycle affect brain excitability and pain pathways^6,7^, but exactly how these changes lead to worse migraine attacks is still unclear. In this study, we focus specifically on menstrual-related episodic migraine in women.

Most neuroimaging studies of migraine have focused on either functional connectivity in isolation^8–10^ or on structural architecture alone^11–16^. More recently, several works have examined structure–function coupling^17–19^, offering a clearer understanding of how brain anatomy constrains neural function and, in the context of migraine, providing evidence for altered interactions between anatomical pathways and functional networks^20–22^.

Importantly, and more specifically, structural–functional coupling has also been investigated in menstrual migraine, linking structural alterations and functional connectivity to pain in menstrual migraine^23,24^. In general, these studies indicate disrupted connectivity among core hubs and long-range pathways in migraine^25^. However, it remains unclear which information about dysfunction is best captured by specific scales of anatomical communication, an open question we address here using a spectral mapping framework^17–19^.

We investigate the role of direct and indirect anatomical connections in migraine patients compared with healthy controls. Direct connections correspond to direct links identified through MRI tractography, whereas indirect connections capture indirect communication that traverses multiple intermediate regions of the structural connectome. Prior work has established that migraine is associated with abnormal rich-club organization and impaired structural connectivity among hub regions^25^. Because hub regions serve as critical relay stations for indirect communication, their disruption would be expected to disproportionately affect indirect anatomical walks, which traverse multiple intermediate nodes, rather than direct connections, which depend on a single structural link. This reasoning is further supported by structure–function coupling studies demonstrating that indirect anatomical communication systematically shapes functional connectivity beyond direct connections^18,26,27^, and that both group-common and individual-specific coupling patterns can be captured through graph-based approaches^28,29^. Accordingly, we hypothesize that menstrual migraine-related differences in structure–function mapping will be most evident at indirect communication scales rather than at the level of direct structural links.

To test this, we apply a spectral-graph mapping framework^17,18,30^ that decomposes communication by walk length in the structural connectome, thereby quantifying the contributions of direct and indirect anatomical walks to network function. This framework builds on and extends previous approaches^17,18,30^ by incorporating recent methodological advances that allow us to isolate communication scales with precision. Beyond quantification, we further localize these alterations across the brain using a state-of-the-art correspondence approach^31^, providing spatial specificity to our conclusions.

## Materials

### Dataset

Data for this study were drawn from the MIG_N2Treat cohort^19,32–36^, comprising 18 female patients diagnosed with episodic migraine without aura, according to the ICHD-III criteria^37^, and 16 age- and gender-matched healthy controls. All patients experienced menstrual or menstrually-related episodic migraine, in line with the design of the cohort^19,32–36^. For each participant, DWI and resting-state fMRI data were acquired and preprocessed as described in the aforementioned studies. A complete description of recruitment procedures, inclusion and exclusion criteria, and clinical assessments is available in these previous publications.

**Fig. 1:**
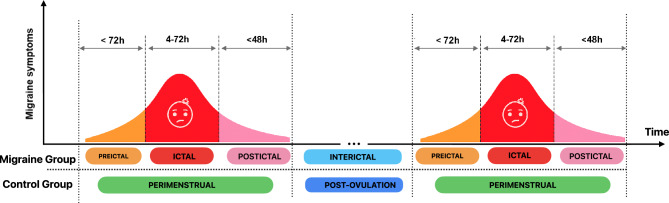
Schematic of the *MigN2treat*scanning protocol, based on Esteves et al.^33^. The vertical axis represents migraine symptom intensity, while the horizontal axis represents time. For migraine patients (top row), four sessions are depicted: preictal (orange; less than 72 hours before menses onset), ictal (red; 4–72 hours after migraine onset), postictal (pink; less than 48 hours after the attack), and interictal (light blue; post-ovulation). Symptom progression is illustrated by a bell-shaped curve that peaks during the ictal phase. For healthy controls (bottom row), two sessions are aligned with patient phases: perimenstrual (green; approximately five days before or after menses), which aligns with preictal, ictal, and postictal phases; and post-ovulation (dark blue; around day 19 of the cycle), corresponding to the interictal phase.

In this study, data were acquired across four MRI sessions (see Fig. 1) aligned with distinct migraine phases: preictal (<72 h before the next attack), ictal (4–72 h after attack onset), postictal (<48 h after attack resolution), and interictal. The perimenstrual period comprised the preictal, ictal, and postictal phases, whereas the post-ovulation period corresponded to the interictal phase. Healthy controls underwent two MRI sessions matched to these menstrual phases: perimenstrual (approximately five days before to five days after menses) and post-ovulation (around day 19 of the menstrual cycle)^32^.

For the present analysis, we included only interictal scans in migraine patients (acquired at least 48 h after the last attack) and post-ovulation scans in controls (day 19), thereby harmonizing measurement timing across groups (see Fig. 1). Data acquired during menses were excluded due to strong evidence that this phase is associated with structural and functional brain changes that could confound the results^38–42^. Of the 16 controls originally enrolled, 15 completed the post-ovulation session. In the patient group, 18 were initially included, and 14 completed the interictal session.

All participants were native Portuguese speakers, aged 18–55 years, with at least nine years of formal education. Exclusion criteria at recruitment included any neurological disorder (apart from migraine in the patient group); current psychiatric illness (based on the State–Trait Anxiety Inventory and Zung Depression Scale); daily use of psychoactive medications (including migraine prophylactics); hormonal or reproductive conditions disrupting cyclic menses (e.g., pregnancy, breastfeeding, post-menopause, or continuous contraception); and standard MRI contraindications. After enrollment, participants with incidental brain lesions detected on T1-weighted scans were excluded from the final analysis. One healthy control was excluded for an incidental finding, and another due to technical failure during data acquisition. Recorded clinical variables for patients included: age (years), migraine onset (age in years at which the participant first experienced migraine meeting diagnostic criteria), disease duration (time in years living with migraine) and monthly attack frequency (migraine attacks per month). Neuroimaging was performed using a Siemens Vida 3 T scanner equipped with a 64-channel head coil, between June 2019 and November 2022^32^. Foam padding and earplugs were used to minimize head motion and reduce scanner noise.

All participants provided written informed consent, and the study protocol was approved by the Ethics Committee of Hospital da Luz, Lisbon, Portugal,(Ref. CES/46/2018/ME) in accordance with the Declaration of Helsinki.

### Structural data acquisition and preprocessing

DWI was performed using a multi-shell echo-planar imaging sequence (repetition time (TR) = 6800 ms; echo time (TE) = 89 ms; flip angle = $90^{\circ }$) with 66 contiguous axial slices at 2 mm isotropic resolution. Acceleration was achieved using GRAPPA (factor 2) and SMS excitation factor 3. Three non-zero b-values (400, 1000, and 2000 s/mm²) were sampled along 32, 32 and 60 unique gradient directions, respectively, along with eight $b_0$ volumes and three additional reverse-phase-encoded $b_0$ images (posterior to anterior), resulting in a total DWI scan time of approximately 15 min 47 s.

High-resolution T1-weighted anatomical images were acquired using a 3D MPRAGE sequence (TR = 2300 ms; TE = 2.98 ms; time of inversion (TI) = 900 ms; flip angle = $9^{\circ }$; 1 mm isotropic voxels; FOV = $256\times 240\times 128$ mm³) in a 5 min 12 s protocol. A sagittal FLAIR acquisition (TR = 5000 ms; TE = 386 ms; TI = 1800 ms; 0.9 mm isotropic resolution; FOV = $240\times 240\times 151$ mm³; GRAPPA = 2) was also obtained in 5 min 57 s for white-matter lesion screening. All structural images were visually inspected by a board-certified neuroradiologist prior to further analysis.

Preprocessing of DWI data followed validated procedures in the MigN2treat cohort^32,33^. Image processing was conducted using FSL^43^ (v6.0.5) and MRtrix3^44^ (v3.0.3), following the DESIGNER^45^ pipeline. Briefly, diffusion data were denoised (dwidenoise) and corrected for Gibbs ringing (mrdegibbs)^46^ and bias-field inhomogeneity (dwibiascorrect with the-ants option) in MRtrix3, while susceptibility-induced distortions, eddy-current distortions, and head motion were corrected using FSL (topup/eddy)^43^. Fiber orientation distributions were estimated using multi-tissue constrained spherical deconvolution, intensity-normalized across subjects, and employed for anatomically constrained probabilistic tractography^32,47–49^. Tractograms were subsequently filtered to reduce reconstruction bias. No log-transformation was applied. Further implementation details are provided in Matoso et al.^32^.

### Functional data acquisition and preprocessing

Resting-state functional MRI data were acquired during the same scanning sessions as the structural data^33^. A T2-weighted gradient-echo echo-planar imaging sequence was used with the following parameters: TR = 1260 ms, TE = 30 ms, flip angle = $70^{\circ }$, GRAPPA acceleration factor = 2, simultaneous multi-slice (SMS) factor = 3, and 60 contiguous axial slices with 2.2 mm isotropic resolution. Each participant completed a 7-minute resting-state scan (333 volumes) during which they fixated on a black screen with eyes open and were instructed to remain still and let their thoughts wander freely.

To correct for B0 field inhomogeneities, dual-echo gradient-echo field maps were also acquired (TR = 400 ms; TE = 4.92/7.38 ms; flip angle = $60^{\circ }$; voxel size = $3.4 \times 3.4 \times 3.0$ mm³). All images were visually inspected for artifacts prior to preprocessing.

Functional MRI preprocessing was performed using FSL^43,50^ tools (e.g., BET/FAST/FLIRT/FNIRT and related utilities), following the validated MigN2treat pipeline described in Esteves et al.^33^.Preprocessing included fieldmap-based distortion correction, motion realignment, high-pass temporal filtering, and nuisance regression (motion parameters, physiological signals, and motion outliers), followed by light spatial smoothing. Regional blood-oxygenation-level–dependent (BOLD) time series were then extracted for each ROI to construct functional connectivity matrices. Further methodological details are available in Esteves et al.^33^.

## Methodology

### General pipeline

**Fig. 2:**
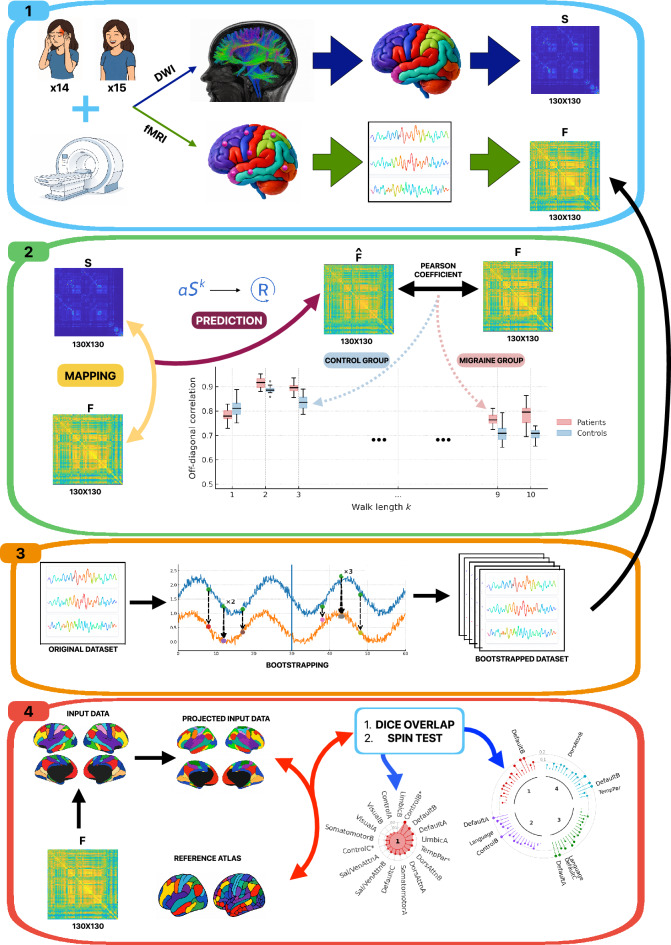
Pipeline Overview. (1) Inputs and matrices. We scanned 14 migraine patients and 15 healthy controls. Preprocessing yields a structural connectivity matrix $S \in \mathbb {R}^{130 \times 130}$ from DWI tractography and a functional connectivity matrix $F \in \mathbb {R}^{130 \times 130}$ from fMRI, both based on a 130-ROI parcellation. **(2) Spectral mapping and prediction.** For each walk length $k \in \mathbb {N}$, where *k* denotes the number of steps in a structural walk, structural and functional eigenmodes are aligned via a rotation matrix $R \in \textrm{SO}(130)$. The contribution of walks of length *k* is modeled using $S^k$, the *k*-th power of the structural matrix. We quantify accuracy as the off-diagonal Pearson correlation between the predicted matrix $\hat{F} \in \mathbb {R}^{130 \times 130}$ and the empirical functional matrix $F \in \mathbb {R}^{130 \times 130}$. **(3) Validation.** The full pipeline is repeated on nonparametric bootstrap resamples of the BOLD time series. The blue trace represents the original BOLD time series, and the orange trace represents an example bootstrap-resampled surrogate. The symbols $\times 2$ and $\times 3$ indicate repeated resampling to generate multiple surrogate time-series realizations for robustness assessment. We compare patient–control distributions across *k* using Kolmogorov–Smirnov tests. **(4) Spatial correspondence.** Input maps are projected onto the cortical surface and compared with a reference network atlas. We assess spatial correspondence via Dice overlap and validate results using spin tests.

This section provides an overview of the analysis pipeline, which is detailed in the subsequent subsections. Figure 2 summarizes the main workflow.

For each participant, DWI and fMRI data were preprocessed to construct a structural connectivity matrix $S \in \mathbb {R}^{130 \times 130}$ and a functional connectivity matrix $F \in \mathbb {R}^{130 \times 130}$, based on 130 regions of interest (ROIs) (see “Connectivity Matrices”). The functional matrices were derived from blood-oxygenation-level-dependent (BOLD) time series extracted from each ROI.

After constructing $S \in \mathbb {R}^{130 \times 130}$ and $F \in \mathbb {R}^{130 \times 130}$, we mapped how structural walks of varying lengths shape functional connectivity using a spectral graph mapping approach, generating a predicted matrix $\hat{F} \in \mathbb {R}^{130 \times 130}$ (see “Individual Contributions of Walk Lengths”). Prediction accuracy was quantified per subject as the Pearson correlation between $\hat{F}$ and the empirical $F \in \mathbb {R}^{130 \times 130}$ (see “Correlation Metrics”).

To assess stability, we repeated the analysis on nonparametric and block bootstrap resamples of the BOLD data, and compared patient-control distributions across walk lengths using Kolmogorov-Smirnov tests (see “Bootstrap Validation and Robustness”).

Finally, we localized group-level effects by projecting degree maps onto the cortical surface and quantifying their spatial correspondence with canonical network atlases using the Network Correspondence Toolbox^31^ (see “Spatial Localization of Network Alterations in Migraine Patients”).

### Connectivity matrices

Both structural and functional connectivity matrices comprise 130 ROIs, obtained by combining 100 cortical parcels from the Schaefer atlas^51^ with all 30 subcortical and cerebellar parcels from AAL116^32,33^. Inclusion of subcortical and cerebellar regions is motivated by their documented involvement in migraine^52^. The complete list of ROIs is provided in the Supplementary Information (see sub-section “Anatomical Parcelation and Recording Sites”).

**Fig. 3:**
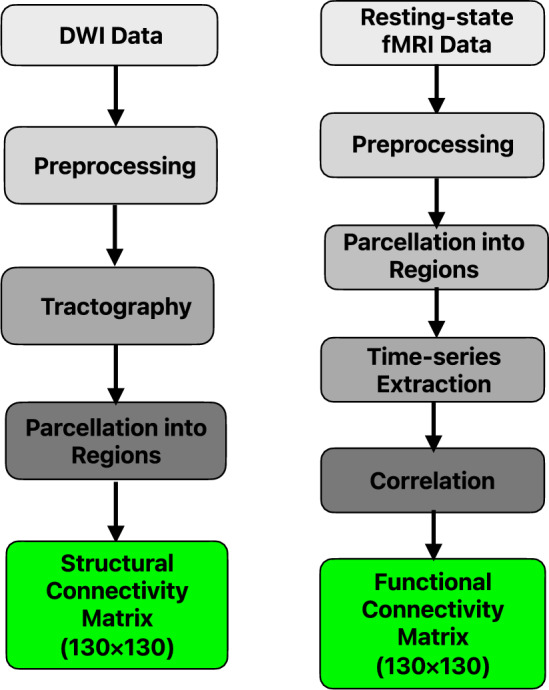
Pipeline for constructing and comparing structural and functional brain networks, as previously performed and reported in^32,33^ and adapted from Sporns (2014) ^53^. The left column shows the structural pipeline. The process begins with preprocessing DWI data, followed by whole-brain probabilistic tractography. The resulting tractogram is parcellated into 130 anatomical regions, and tractography-derived inter-regional connection weights define the structural connectivity matrix $S \in \mathbb {R}^{130 \times 130}$. The right column presents the functional pipeline. Resting-state fMRI data was preprocessed and parcellated into the same 130 regions used in the structural pipeline, by extracting the mean BOLD time series from each ROI. Pearson correlations between regional time series are computed to obtain the functional connectivity matrix $F \in \mathbb {R}^{130 \times 130}$.

Structural $S \in \mathbb {R}^{130\times 130}$ and functional $F \in \mathbb {R}^{130\times 130}$ connectivity matrices were derived from the imaging data detailed in the Materials section and used as inputs for all subsequent graph-theoretic analyses. Figure 3 summarizes the pipeline for constructing both matrices.

#### Structural connectivity matrix

Structural connectivity matrices play a fundamental role in mapping brain network architecture and provide the basis for graph-theoretical and spectral analyses.

As reported in Matoso et al.^32^, structural connectivity matrices were constructed from tractography results as weighted, undirected connectomes. For each pair of ROIs (*i*, *j*), the corresponding edge weight was computed as the sum of the streamline weights of all streamlines connecting the two regions. These connection strengths were further scaled using inverse node volume normalization, rather than relying on raw streamline counts alone. Because diffusion-weighted imaging tractography does not provide reliable information about anatomical directionality, the structural connectivity matrix $S \in \mathbb {R}^{130 \times 130}$ was treated as symmetric, such that $S_{ij}=S_{ji}$^32^. Symmetry was enforced during connectome generation in MRtrix3 using the tck2connectome option-symmetric, and diagonal elements, representing self-connections, were set to zero using the-zero_diagonaloption. Further implementation details are provided in Matoso et al.^32^.

#### Functional connectivity matrix

Functional connectivity matrices $F \in [-1, 1]^{130 \times 130}$ quantify statistical dependencies between distinct brain regions based on resting-state fMRI data.

As reported in Esteves et al.^33^, to construct the matrices, preprocessed BOLD signals were averaged within each ROI to obtain representative time series.

Let the time series of ROI *i* and ROI *j* described by,

$$x_i = \begin{bmatrix} x_{i1}, x_{i2}, \ldots , x_{iT} \end{bmatrix}^\top \text {and} \quad x_j = \begin{bmatrix} x_{j1}, x_{j2}, \ldots , x_{jT} \end{bmatrix}^\top \text {respectively,}$$

where *T* denotes the total number of sample points.

These time series were filtered using a second-order Butterworth bandpass filter (0.01–0.1 Hz), retaining low-frequency oscillations commonly associated with functional connectivity.

The functional connectivity between regions *i* and *j* is defined by the Pearson correlation $\rho :\mathbb {R}^T \times \mathbb {R}^T$ with values in $[-1,1]$, applied to their BOLD time series $x_i, x_j \in \mathbb {R}^T$. The resulting value $\rho (x_i, x_j)$ populates the off-diagonal entries of *F*.

The diagonal entries are set to 1, reflecting perfect self-correlation. All *F* matrices are symmetric, since $\rho (x_i, x_j) = \rho (x_j, x_i)$. Positive values indicate synchronous (positively correlated) activity, negative values indicate anti-correlation, and values near zero suggest weak or no linear association.

#### Individual contributions of walk lengths

In the present work, to predict the functional connectivity matrix $\hat{F} \in \mathbb {R}^{130 \times 130}$ from the structural connectivity matrix $S \in \mathbb {R}^{130 \times 130}$, we employ a spectral-based model based on the previous work done by Becker et. al^18^ that incorporates two key components: eigenmode alignment via a rotation matrix $R \in \mathbb {R}^{130 \times 130}$, which aligns the eigenvectors of $S \in \mathbb {R}^{130 \times 130}$, with those of the empirical functional matrix $F \in \mathbb {R}^{130 \times 130}$, and polynomial contributions associated with the powers of the adjacency matrix of the structural graph, the matrix $S \in \mathbb {R}^{130 \times 130}$, that reflect structural walks of length $k$.

This approach builds on the framework of spectral graph theory, where the powers of $S \in \mathbb {R}^{130 \times 130}$ encode both direct and indirect anatomical communication through the network. Each power $S^k$ corresponds to walks of length $k$, with longer walks capturing increasingly complex indirect interactions across the brain’s anatomical structure (see Supplementary Information).

Our method extends the spectral mapping model originally proposed by Becker et al.^18^, which estimates functional connectivity as a cumulative sum of structural walk contributions up to a given length $k$. In contrast, we isolate the individual predictive contribution of each walk length, enabling a more detailed analysis of how distinct indirect pathways relate to emergent functional organization.

The predicted functional matrix is given by

1 $$\begin{aligned} \hat{F} = R \left( a_0I +a_k S^k \right) R^\top , \end{aligned}$$

where the coefficients $a_0, \dots , a_k \in \mathbb {R}$ weight the contributions of walks of different lengths.

When $k = 0$, the model includes only the identity term $S^0 = I$, representing intrinsic activity that is independent of network interactions. For $k = 1$, the model accounts for direct anatomical connections via $S^1$, enabling signal propagation between directly connected ROIs. At $k = 2$, the term $S^2$ captures interactions mediated by a single intermediate region. More generally, for any $k \in \mathbb {N}$, the model incorporates walks of lengths $k$, accounting for increasingly complex indirect interactions.

The individual contribution of walk length $k$ is evaluated by solving

2 $$\begin{aligned} \min _{R,\,a_0,\,a_k} \left\Vert F - R \left( a_0 I + a_k S^{k} \right) R^{\top } \right\Vert _F^{2}, \qquad \text {subject to } R^{\top } R = I, \end{aligned}$$

where $R \in \textrm{SO}(130)$ is the rotation matrix aligning the structural and functional eigen-bases.

The constrained optimization problem takes place on the Riemannian manifold of special orthogonal matrices, ensuring that *R*remains orthogonal throughout the process. Unlike traditional Euclidean methods, this approach preserves the geometry of the eigenbasis alignment and is well-suited to the nonlinear structure-function transformation being modeled^54^. This formulation preserves the global baseline $a_0 I$ while isolating the effect of the single walk term $S^{k}$. The optimization is performed independently for each $k$ and the resulting prediction $\hat{F}$ is then compared to the empirical $F$ via Pearson correlation, yielding a score $r_k$ that reflects how much variance in functional connectivity is explained by walks of length $k$. By computing $r_k$ across $k$ for both migraine patients and healthy controls, we directly compare the predictive contributions of direct (small $k$) versus indirect (large $k$) structural pathways, thereby testing whether menstrual migraine shifts the balance of functional reliance on anatomical connections relative to healthy controls.

### Correlation metrics

Reconstruction accuracy was quantified as the Pearson correlation between the vectorized off-diagonal entries of $\hat{F}$ and *F*. Let $n \in \mathbb {N}$ be the number of ROIs. There are $m = n(n - 1)/2$ off-diagonal entries in each symmetric matrix. We extract these entries into two vectors of length *m*, one from *F* and one from $\hat{F}$, center each vector by subtracting its mean, and compute the Pearson coefficient.

The scalar correlation coefficient $\rho \in [-1,1]$ provides a single measure of overall pattern similarity between the predicted and empirical functional connectivity matrices. Values near $+1$ indicate a positive correlation between the predicted matrix and the empirical one, values around 0 imply no systematic correspondence, and negative values reflect an inverse relationship between the two matrices.

#### Bootstrap validation and robustness

To increase statistical robustness and assess the stability of the KS test results, we employed a non-parametric bootstrapping approach. For each subject’s preprocessed BOLD time series, 10 resampled surrogates were generated, producing a bootstrap dataset that captures variability in the functional connectivity estimates. The full bootstrap results are reported in the Supplementary Information (Section 8.1), including the two-sided KS bootstrap p-value distributions across walk lengths (Figure 11 from Supplementary Information).

In addition to the standard bootstrap, we implemented a block bootstrap procedure with a block size of 5 time points to account for temporal autocorrelation in the BOLD signal. Given the limited series length (333 samples), this block size increases the number of effective resampling units while still partially preserving autocorrelation. Block bootstrap validation is also reported in Supplementary Information (Section 8.1.3).

###  Spatial localization of network alterations in migraine patients

Understanding where migraine-related alterations emerge is as important as quantifying what changes occur. We tried to localize migraine-related network changes by capturing two complementary aspects of large-scale organization: the magnitude and the statistical significance of overlaps between ROI-level connectivity and canonical networks. By quantifying both, we identify which networks are most affected, understanding organizational shifts that may underlie migraine pathology.

To this end, we employed the CBIG Network Correspondence Toolbox (NCT)^31^ to generate and statistically compare intrinsic connectivity (degree) maps against reference atlas. Input maps were derived from all 130 ROIs of our parcellation, however, because the reference atlases are cortical-only, only the 100 cortical regions contributed directly to the overlap computations.

First, we computed group-mean functional degree maps for patients and controls by summing each ROI’s functional connections and projecting the resulting 130-element vectors back onto our combined atlas. Specifically, degree was computed as signed functional node strength, defined as the sum of raw Pearson correlation coefficients across all connections of a node; thus, positive and negative correlations contribute with opposite sign. Because this scalar summary collapses the full edge-weight distribution into a single value, we interpret these maps as a coarse summary of overall nodal connectivity rather than a complete characterization of functional coupling.

For the correspondence analysis, we retained the top 25% most-connected ROIs and measured the proportion assigned to each canonical network, then we quantified the spatial overlap between high-degree regions and these networks using NCT’s spatial-overlap workflow^31^. NCT takes your thresholded cortical surface map and the chosen atlas networks, resamples the atlas labels onto the same surface mesh as your map, and then computes surface-based Dice overlap and spin-test statistics. The Dice coefficient quantifies spatial overlap between two binary maps on a 0–1 scale (0 indicates no overlap; 1 indicates perfect agreement). Here, one map is the thresholded cortical map and the other is the canonical network mask from the reference atlas. To ensure robustness across parcellation, this procedure was repeated for four widely adopted Schaefer-based atlases in migraine research^55,56^:

1. **AS400Y17:** Schaefer-400 parcels with Yeo-2011 17-network labels,
2. **AS400K17:** Schaefer-400 parcels with Kong-2021 17-network labels,
3. **AS200K17:** Schaefer-200 parcels with Kong-2021 17-network labels, and
4. **AS200Y17:** Schaefer-200 parcels with Yeo-2011 17-network labels.

This quantifies the relative importance of each network in migraine patients and healthy controls. To evaluate statistical significance of each hub–network overlap, we computed empirical $p$–values by comparing the observed overlap against a null distribution obtained from hemisphere-preserving random rotations of the cortical maps (spin tests), thereby assessing whether overlaps of equal or greater magnitude could arise by chance.

## Results

We divide our results into two parts. First, we report the structure–function mapping results obtained by isolating the contribution of individual walk lengths. Second, we examine the anatomical localization of connectivity alterations and evaluate their spatial correspondence with established reference atlases.

### Individual walk–length contributions to structure–function mapping

**Fig. 4:**
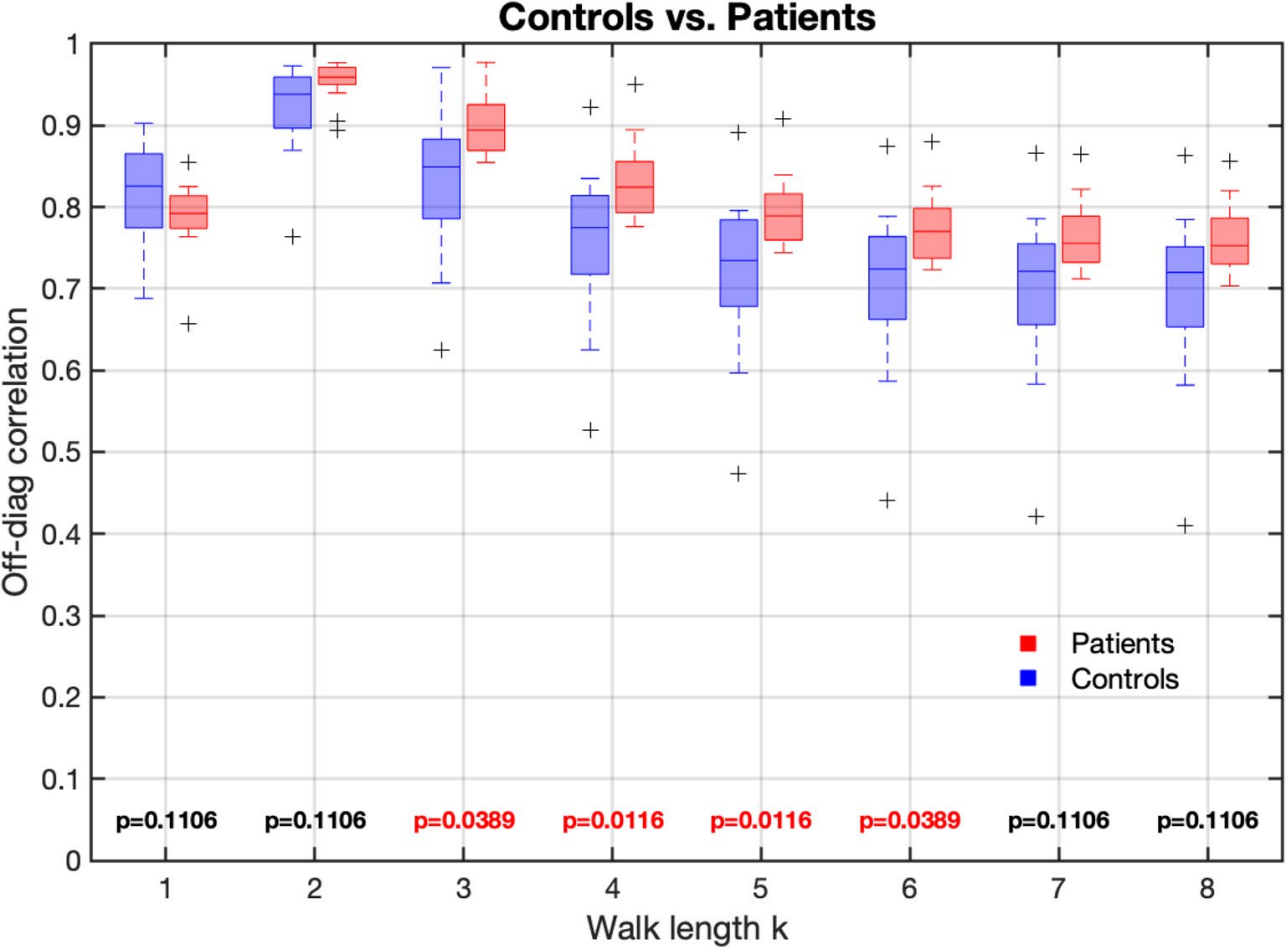
Boxplots show the distribution of Pearson correlation coefficients between predicted and empirical functional connectivity matrices based on individual walk contributions for lengths $k=1$ to $k=8$. These values reflect in-sample spectral reconstruction after optimizing the alignment and coefficients using the same subject functional connectivity and should not be interpreted as out-of-sample prediction performance. Blue boxes correspond to controls and red boxes to patients; central marks indicate medians, box edges the interquartile range (IQR), whiskers 1.5$\times$ the IQR, and crosses outliers. Raw two-sample KS *p*-values are displayed below each pair of boxplots, with red indicating uncorrected $p<0.05$. After Benjamini–Hochberg FDR correction across walk lengths ($q=0.05$), only $k=4$ ($p_{\textrm{FDR}}=0.046$) and $k=5$ ($p_{\textrm{FDR}}=0.046$) remained significant.

Figure 4 shows the estimated contribution of each walk length.

When we isolate the contribution of structural walks of exactly length $k$ to the prediction of the functional matrix $\hat{F}$, the mapping accuracy varies non-monotonically with $k$ (note that the results show the contribution of each walk length individually, rather than their cumulative contribution). We restricted the analysis to $1\le k\le 8$ because cumulative mapping performance plateaus at $k\approx 8$^18^, and at larger *k* the matrices $S^{k}$ become increasingly collinear with lower powers, making walk-length-specific effects harder to identify (see Supplementary Information). Using only direct paths ($k = 1$) yields a median Pearson correlation of 0.8185 in controls (IQR = 0.0831) and 0.7885 in migraine patients (IQR = 0.0400). The two-sample KS test yields $p = 0.1106$, indicating no statistically significant group difference at this first-order level.

Two-step walks ($k = 2$) correspond to contributions mediated by a single intermediate region. In this case, controls exhibit a median correlation of 0.9194 (IQR = 0.0633) and patients 0.9535 (IQR = 0.0209), with a group comparison again yielding $p = 0.1106$. Two-step walks ($k = 2$) capture nearly the full structure–function relationship, and do so with reduced inter-subject variability, particularly in the patient group.

At $k = 3$, a moderate decline in correlation is observed (controls: 0.8327, IQR = 0.0975; patients: 0.9007, IQR = 0.0561), accompanied by an uncorrected significant difference ($p = 0.0389$), which did not survive FDR correction ($p_{\textrm{FDR}} = 0.078$). Statistically significant group differences emerge at intermediate walk lengths: for instance, $k = 4$ yields $p = 0.0116$ (corrected $p_{\textrm{FDR}} = 0.046$), with median correlations of 0.7584 (IQR = 0.1061) in controls and 0.8329 (IQR = 0.0625) in patients. Similarly, at $k = 5$, the difference remains significant ($p = 0.0116$, $p_{\textrm{FDR}} = 0.046$), with medians of 0.7231 (IQR = 0.1058) versus 0.7949 (IQR = 0.0567)).

A comparable trend is observed for $k = 6$, where the uncorrected $p = 0.0389$ (corrected $p_{\textrm{FDR}} = 0.078$), and median correlations reach 0.7063 (IQR = 0.1010) and 0.7747 (IQR = 0.0611) for controls and patients, respectively. These findings suggest that structural walks of length 4 and 5 capture subtle yet reproducible group-specific information in structure–function coupling.

For longer walks ($k \ge 7$), mapping accuracy stabilizes, with median correlations converging within 0.698-0.695.698.695 in controls and 0.765-0.759.765.759 in patients, and all $p$-values exceeding 0.05. The IQR also decreases substantially (e.g., $k = 8$: IQR 0.0980 vs. 0.0562), indicating that longer walks yield minimal and highly homogeneous contributions in both groups.

In summary, two-step paths dominate predictive accuracy. Intermediate walk lengths ($k\in 4,5$) introduce statistically modest but significant group differences, whereas very short or very long walks do not.

#### Bootstrap validation and robustness

The bootstrap results indicate that the significance of group differences varies systematically with walk length. Specifically, walk lengths $k\in [3,5]$ yield consistently median $p$-values across bootstrap replicates below the $\alpha = 0.05$ threshold. This suggests that the observed differences between patient and control distributions at these intermediate walk lengths are robust to sampling variability.

In contrast, the distributions of $p$-values for other walk lengths, particularly $k\in 1,2$ and $k \ge 6$, are broader and centered above $\alpha = 0.05$, indicating that group differences at those scales are either weaker or less stable across bootstraps. These findings reinforce our earlier conclusion that intermediate-length indirect walks of lengths $k\in 4,5$, carry the most consistent and statistically reliable discriminative information.

Analogously to the analysis performed on the original dataset, we subjected the bootstrap-derived dataset to a KS test. The results, shown in the Supplementary Information, are consistent with those obtained from the empirical data, supporting the robustness of the observed group differences across walk lengths $k\in 4,5$.

#### Block bootstrap validation and robustness

Furthermore, we considered block bootstrapping to account for the temporal autocorrelation inherent in BOLD time series. With 333 time points, we used blocks of 5 samples, which increases the number of effective resampling units while still partially preserving autocorrelation structure. Although this block size cannot fully capture longer correlation ranges, the resulting surrogate datasets provide a more principled control than standard bootstrapping. Importantly, the block bootstrap results were qualitatively consistent with those from the standard bootstrap analysis (see Supplementary Information), reinforcing the robustness of the observed group-level differences across walk lengths.

### Computing the spatial localization of network-level alterations

**Fig. 5:**
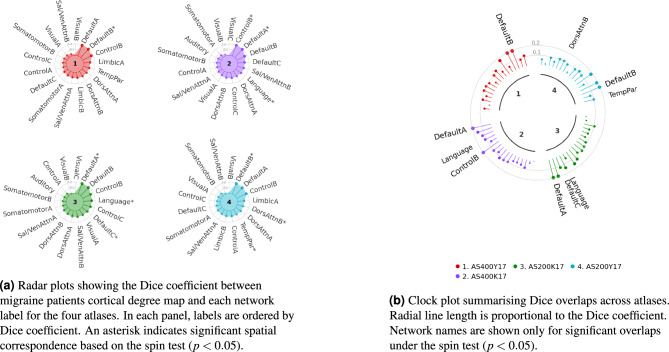
Graphical overview of NCT-derived spatial correspondence between the cortical degree map and canonical networks in patients with migraine.

By comparing overlap metrics between patients degree maps and atlas for each network, we identified which brain networks show the most pronounced functional differences in menstrual migraine. Figure 5 shows the results obtained.

**Fig. 6:**
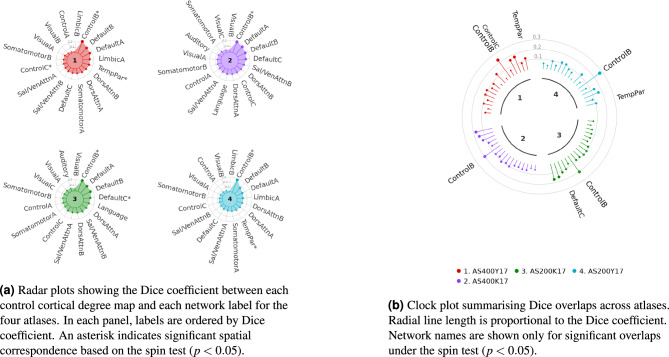
Graphical overview of cortical degree map to network spatial correspondence obtained with NCT in the control group.

Dice coefficients and associated spin-test *p*-values are listed in the Supplementary Information. Significant overlaps ($p<0.05$) appear most consistently in the *DefaultA* and *DefaultB* networks across all four atlases. The strongest match occurs for *DefaultA* in AS200K17 (Dice = 0.1921, $p=0.025$), followed by *DefaultB* in AS400Y17 (Dice = 0.1974, $p=0.031$).

To determine the specificity of these findings, we repeated the same analysis in the healthy control group. Figure 6 presents the results.

In the results obtained some overlap with default-mode and control networks was also present, the effect sizes were generally lower and statistical significance less consistent (See Supplementary Information for a more detailed description), suggesting that the spatial engagement of canonical networks observed in the migraine group is not simply a generic feature of brain connectivity.

## Discussion

We provide converging evidence that migraine selectively alters the mesoscale structure–function mapping of the brain, increasing the degree to which indirect anatomical pathways constrain resting-state dynamics. Together, these findings indicate that menstrual migraine is characterized by a scale-specific reconfiguration of structure–function coupling, with the strongest group differences emerging at intermediate indirect walk lengths ($k\in 4,5$). We now discuss the implications of these findings by organizing the discussion into four parts.

### Cumulative versus individual walk contributions

Our analysis confirms the canonical principle by Beckeret al.^18^: structural walks up to length three suffice to reconstruct large-scale functional networks with near-perfect fidelity, and mapping accuracy saturates for $k> 7$. We replicate this result in both groups, with cumulative models yielding over 99% correlation. However, these cumulative models proved to be non-informative for distinguishing between groups (see Supplementary Information), suggesting that traditional global mappings obscure clinically relevant signal.

To address this, we assessed the individual predictive contributions of each walk length independently. As shown in Fig. 4, a non-monotonic group dissociation emerged. At short walk lengths ($k = 1$), control participants showed marginally higher alignment than migraine patients ($p = 0.1106$), consistent with preserved direct integrity. By contrast, at intermediate lengths ($k\in [3,6]$), migraine patients showed significantly higher structure–function alignment than controls ($p=0.0389, 0.0116, 0.0116,$ and 0.0389, respectively), indicating stronger coupling between functional connectivity and indirect structural routes at these mesoscale levels. The effects observed at intermediate walk lengths ($k\in 4,5$) survived FDR correction and remained robust under extensive bootstrap resampling (see Supplementary Information), confirming their statistical reliability. Notably, for both very short and long walks ($k < 3$ and $k> 7$), group differences were attenuated, reinforcing the specificity of this mesoscale effect.

### Spatial specificity of network alterations

To anatomically contextualize these spectral alterations, we employed the NCT^31^, comparing functional degree maps against canonical large-scale network templates. We found that in patients, hub-related deviations were spatially concentrated within the Default Mode Network, particularly the DefaultA and DefaultB subsystems. This fingerprint was significantly less pronounced in controls, suggesting a disease-specific shift in integrative dynamics. Notably, this aligns with prior observations of increased intrinsic Default Mode Network connectivity in migraine without aura^57,58^, as well as disruptions in limbic hubs described in connectome fingerprinting analyses^33^.

### Methodological advances and limitations

Our framework contributes with two methodological innovations. First, we integrate Riemannian optimization over the orthogonal group, ensuring that the eigenbasis alignment transformation remains orthogonally constrained and geometrically faithful, this improves upon previous spectral approaches that relied on approximated alignments. Second, by separating cumulative and individual walk-length contributions, we uncover clinically meaningful effects that remain hidden in conventional models^59^.

Nonetheless, several limitations must be acknowledged. The cohort is relatively small, exclusively female, and restricted to episodic migraine without aura, limiting generalizability. Diffusion tractography underestimates long-range fibers^60,61^ and in both matrices there is a lack of directionality, potentially biasing walk-based estimations. Moreover, while our parcellation included 130 regions (100 cortical, 30 subcortical/cerebellar), the Network Correspondence Toolbox relies on cortical surface templates, meaning that only the cortical ROIs contributed directly to the spatial correspondence analyses. Accordingly, because degree was computed as signed node strength, our correspondence analysis is intended to localize broad network-level involvement, whereas sign-specific mechanisms require dedicated analyses beyond the scope of the present work. We also treat the structural connectome as fixed ground truth, ignoring possible dynamic plasticity^35,62,63^. From a computational perspective, the underlying optimization problem is inherently non-convex, however, we implemented multiple initializations and evaluated convergence consistency across iterations to ensure near-optimality (see Supplementary Information).

Additionally, the interpretation of higher-order walk lengths should be made with caution. Because higher powers of the structural matrix become increasingly collinear with lower powers, the individual walk-length effects are harder to uniquely identify at large *k*. Moreover, the reported correlations reflect in-sample spectral reconstruction, in which both the rotation matrix and polynomial coefficients are optimized using the same subject’s empirical functional connectivity, rather than out-of-sample prediction. Consequently, these values should not be compared directly with predictive benchmarks from cross-validated or held-out frameworks^27–29^.

### Outlook and broader applicability

Beyond migraine, the methodology presented here offers a generalizable framework for probing structure–function relationships in neurological disease. The ability to pinpoint walk lengths that are differentially affected provides a method to study disease mechanisms. Future applications may leverage these insights to guide network-informed interventions, including neuromodulatory or pharmacological strategies aimed at restoring functional balance by modulating communication along specific anatomical pathways. In the context of migraine, our findings raise the possibility of therapeutic targeting of intermediate walk-length connectivity, potentially offering a path to symptom reduction or attack prevention through connectome-informed strategies.

## Supplementary Information

Supplementary Information.

## Data Availability

Example structural and functional connectivity matrices that allow replication of the main analyses are available in the Spectral Connectivity repository: https://github.com/ggoncalo02/SpectralConnectivity. The full neuroimaging dataset analysed during the current study is not publicly available due to patient privacy restrictions but is available from the corresponding author on reasonable request.
