# Supplementary material for: Integrated anatomical and functional connectivity mapping in episodic migraine: a spectral graph theory approach
Source: Sci Rep. 2026 May 20;16:22871. doi: 10.1038/s41598-026-53726-7 (PMC13388938; doi:10.1038/s41598-026-53726-7)
Supplement: Supplementary file 1 — Supplementary Information. [file 41598_2026_53726_MOESM1_ESM.pdf]

Supplementary Information for "Integrated Anatomical and Functional Connectivity Mapping in Episodic Migraine: A Spectral Graph Theory Approach"

Gonçalo Grácio[1,*], Ana Matoso[2], Inês Esteves[2], Ana R. Fouto[2], Amparo Ruiz-Tagle[2], Gina Caetano[2], Raquel Gil-Gouveia[3,4], Patrícia Figueiredo[2], Rita G. Nunes[2], and Sérgio Pequito[1]

[1]Institute for Systems and Robotics – Lisboa and Department of Electrical and Computer Engineering, Instituto Superior Técnico, University of Lisbon, Lisbon,Portugal
[2]Institute for Systems and Robotics – Lisboa and Department of Bioengineering, Instituto Superior Técnico, University of Lisbon, Lisbon,Portugal
[3]Neurology Department, Hospital da Luz, Lisbon, Portugal
[4]Center for Interdisciplinary Research in Health, Universidade Católica Portuguesa, Lisbon, Portugal
[*]goncalo.gracio@tecnico.ulisboa.pt

Contents

1 Overview **2**
2 Spectral mapping: Mathematical Description **2**
2.1 Adjacency matrix and structural walks . . . 2
2.2 Polynomial structure–function mapping . . . 3
2.3 Eigen–decomposition . . . 4
2.4 Rotation Matrix . . . 5
3 Supplementary methods: Cumulative-contributions **8**
3.1 Finding initial values for optimization - Warm-start . . . 8
3.2 Optimisation Process . . . 9
3.3 Implementation details (MANOPT baseline) . . . 10
3.4 Convexity . . . 10
4 Supplementary methods: Individual–contributions **12**
4.1 Spectral least–squares fit on eigenvalues . . . 12
4.2 Rotation step and optimisation problem . . . 12
4.3 Finding initial values for optimisation — Warm-start . . . 12
4.4 Optimisation process . . . 12
5 Spatial Alignment with the Network Correspondence Toolbox **13**
6 Correlation Metrics **14**
7 Perturbation Analysis **16**
8 Supplementary Results **18**
8.1 Bootstrapping . . . 19
Two sided Bootstrap • One sided Bootstrap • Block Bootstrap
9 Supplementary Tables **21**
9.1 NCT Results Tables . . . 22
References **23**

1 Overview

This supplementary information document provides comprehensive mathematical foundations and methodological details for the spectral graph theory approach to analyzing brain connectivity in menstruation-related migraine. The document begins with detailed mathematical descriptions of spectral mapping (Section 2), including adjacency matrices and structural walks (Section 2.1), polynomial structure-function mapping (Section 2.2), eigen-decomposition theory (Section 2.3), and rotation matrix formulations (Section 2.4) that enable proper alignment between structural and functional eigenspaces. Two main methodological approaches are presented: the cumulative-contributions method (Section 3) which examines walks up to length k with warm-start optimization (Section 3.1), Riemannian optimization processes (Section 3.2), MANOPT implementation details (Section 3.3), and convexity analysis proving the non-convex nature of the optimization landscape (Section 3.4); and the individual-contributions method (Section 4) that isolates single walk lengths through spectral least-squares fitting (Section 4.1) and independent rotation optimization (Section 4.2- 4.4). The document includes validation frameworks such as spatial alignment using the Network Correspondence Toolbox for quantifying overlap with canonical brain atlases (Section 5). Comprehensive results are provided including three correlation metrics for performance evaluation (Section 6), and perturbation analysis demonstrating robustness under multiplicative noise (Section 7). Extensive bootstrap validation using one-sided, two-sided, and block bootstrap procedures (Section 8), and complete Network Correspondence Toolbox results tables (Section 9) showing spatial overlap statistics. Supporting materials include detailed anatomical parcellation information for the 130-ROI atlas combining Schaefer-100 and AAL-30 regions, with the methodology demonstrating that structural walks of length 2-3 capture over 95% of structure-function relationships while showing robust performance under realistic data perturbations and revealing consistent group differences between migraine patients and controls.

2 Spectral mapping: Mathematical Description

To predict the functional connectivity matrix $\hat{F} \in \mathbb{R}^{130\times130}$ from the structural connectivity matrix $S \in \mathbb{R}^{130\times130}$, we propose a model that defines $\hat{F}(S)$ as a polynomial transformation of S, aligned through an orthogonal rotation, such that $\hat{F}(S)$ approximates the empirical functional matrix $F \in \mathbb{R}^{130\times130}$ as closely as possible under a similarity metric.

2.1 Adjacency matrix and structural walks

In graph theory terms, the adjacency matrix S of the brain's structural connectome naturally gives rise to the notion of a *walk*: a sequence of regions $(i_0, i_1, \ldots, i_k)$ in which each consecutive pair (i_{k-1}, i_k) is connected. This definition permits repeated visits to the same region. Each region is represented as a node in the structural graph encoded by the matrix S. By contrast, a *path* is a walk in which each node appears at most once. These two concepts are illustrated in Figure 1.

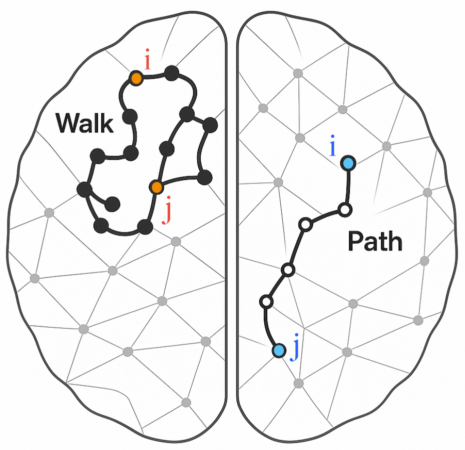

Figure 1. Examples of paths and walks in the human connectome illustrate the numerous communication channels between two arbitrary regions i and j, reflecting core concepts in brain network communication models as discussed by Seguin *et al.*[1].

To explore the structural walks, we consider the graph's adjacency matrix S, an $N \times N$ representation ($N = 130$) in which each off-diagonal entry $S_{ij} \in [0,1]$ denotes the normalized strength of the undirected connection between regions i and j. By construction, S is symmetric ($S_{ij} = S_{ji}$) and has zero diagonal entries ($S_{ii} = 0$), since no region connects to itself. Crucially, successive powers of this matrix S, S^2, S^3, $\dots$ encode walks of increasing length: the entry $[S^k]_{ij}$ aggregates the number and strength of all length-k walks from node i to node j. Analyses of these matrix powers provide direct insight into the brain's polysynaptic structural pathways.

To illustrate this, let the vertex set be

$$V = \{a_1, a_2, a_3, .., a_n\} \tag{1}$$

and define the weighted-adjacency matrix

$$S = \begin{pmatrix} 0 & s_{a_1a_2} & s_{a_1a_3} & \cdots & s_{a_1a_n} \\ s_{a_1a_2} & 0 & s_{a_2a_3} & \cdots & s_{a_2a_n} \\ s_{a_1a_3} & s_{a_2a_3} & 0 & \cdots & s_{a_3a_n} \\ \vdots & \vdots & \vdots & \ddots & \vdots \\ s_{a_1a_n} & s_{a_2a_n} & s_{a_3a_n} & \cdots & 0 \end{pmatrix}, \qquad s_{xy} = s_{yx} \in [0,1],\ s_{xx} = 0. \tag{2}$$

For any vertices $x, y, w \in V$ the (x,y) entry of the square S^2 is

$$(S^2)_{xy} \;=\; \sum_{w \in V} S_{xw} S_{wy}, \tag{3}$$

which equals the total weight of all walks length size 2 from node x to node y that pass through node w. w. If we expand this to S^n instead of S^2, we get the following:

Claim 1. *For every integer $n \geq 1$ and all vertices $x, y, v_i \in V$,*

$$(S^n)_{xy} \;=\; \sum_{v_1 \in V} \sum_{v_2 \in V} \cdots \sum_{v_{n-1} \in V} \left(s_{xv_1} s_{v_1v_2} \cdots s_{v_{n-1}y} \right). \tag{4}$$

The nested sums run over all length-n walks x to v_1 to v_{n-1} to y, and the product multiplies the n corresponding edge-weights.

Proof. **Base case** $n = 1$**.** Since $S^1 = S$, we have $(S)_{xy} = s_{xy}$, the weight of the single edge x to y (or 0 if the edge is absent).

Induction step. Assume the formula holds for some $n \geq 1$. Then

$$(S^{n+1})_{xy} = \sum_{w \in V} (S^n)_{xw} S_{wy}. \tag{5}$$

By the induction hypothesis, $(S^n)_{xw}$ already represents the total weight of all walks of length n from node x to node w. Multiplying by S_{wy} corresponds to adding the final edge from node w to node y, so each product accounts for the weight of a walk from x to y of length $n+1$, passing through w as the penultimate node. Summing over all possible intermediate nodes w thus gives the total weight of all walks of length $n+1$ from x to y.

□

2.2 Polynomial structure–function mapping

The Cayley-Hamilton Theorem states that if $A \in \mathbb{R}^{n \times n}$ is a real symmetric matrix, and $f(\lambda)$ denotes its characteristic polynomial, then the matrix A satisfies its own characteristic equation. Which means, $f(A) = 0$, where,

$$f(\lambda) = \det(\lambda I - A). \tag{6}$$

Writing the characteristic polynomial explicitly as

$$f(\lambda) = \lambda^n + c_{n-1}\lambda^{n-1} + \cdots + c_1\lambda + c_0, \tag{7}$$

the Cayley–Hamilton theorem asserts that

$$f(A) = A^n + c_{n-1}A^{n-1} + \cdots + c_1 A + c_0 I = 0. \tag{8}$$

As a consequence, for any integer $k \geq n$, the power A^k can be expressed as a linear combination of the elements in $\{I, A, A^2, \ldots, A^{n-1}\}$. That is, higher powers of A are redundant and lie in the span of the first n powers.

We have now a polynominal function, $f(\lambda)$, that reconstructs a symetric matrix $n \times n$, in terms (powers) of the A matrix which is exactly what we want. Let $S \in \mathbb{R}^{n\times n}$ denote the structural connectivity matrix of a brain network (symmetric, real), and let $F \in \mathbb{R}^{n\times n}$ be the empirical functional connectivity matrix observed. The goal is to approximate F by a function of S. We can therefore consider an estimated functional connectivity matrix $\hat{F}$ that approximates the empirical matrix F as a linear combination of powers of the structural connectivity matrix S, allowing us to write

$$\hat{F} = \sum_{l=0}^{n-1} a_k S^k. \tag{9}$$

Although an "infinite" power series of S is needed to predict the original F matrix from the S matrix, the Cayley-Hamilton Theorem ensures that for an $n \times n$ matrix, all powers S^k with $k \geq 130$ can be exactly expressed in a linear combination of elements $\{I, S, \ldots, S^{129}\}$. Hence, no further degrees of freedom arise beyond these n coefficients. By Cayley-Hamilton, the empirical functional connectivity matrix F admits an exact (in principle) or low-dimensional (in practice) approximation as a polynomial in the structural matrix S, providing a principled bridge between anatomy and function in brain networks. As we will later see, and in line with previous findings, high-order terms beyond S^7 contribute negligibly to the prediction[2], making such high-degree expansions unnecessary in practice.

2.3 Eigen–decomposition

Eigen-decomposition is a technique that allows to represent a squared matrix in simpler terms.

In our problem, we are dealing with $n \times n$ matrices (squared matrices), in this case, if we consider a non null vector v and a generic symmetric matrix A, if we multiply Av, we are just escalating the vector v, by a constant factor λ. This important relation, can be expressed as:

$$Av = \lambda v. \tag{10}$$

If we try to isolate A, solving (10), we obtain the following:

$$A = V \Lambda V^{-1} = V \Lambda V^{\top}, \tag{11}$$

where

$$\Lambda = \begin{bmatrix} \lambda_1 & 0 & \cdots & 0 \\ 0 & \lambda_2 & \cdots & 0 \\ \vdots & \vdots & \ddots & \vdots \\ 0 & 0 & \cdots & \lambda_n \end{bmatrix}, \tag{12}$$

collects the eigenvalues in descending order, and

$$V = \begin{bmatrix} v_{11} & v_{12} & \cdots & v_{1n} \\ v_{21} & v_{22} & \cdots & v_{2n} \\ \vdots & \vdots & \ddots & \vdots \\ v_{n1} & v_{n2} & \cdots & v_{nn} \end{bmatrix}, \tag{13}$$

contains the corresponding orthonormal eigenvectors.

The decomposition expressed in (11) is significant because it transforms matrix operations into simpler, scalar operations involving eigenvalues, making computations easier.

Because in our problem the structural connectivity S matrix is symmetric, it admits the eigen-decomposition, given by

$$S = V \Lambda V^{-1} = V \Lambda V^{\top}, \tag{14}$$

with

$$\Lambda = \mathrm{diag}(\lambda_1, \ldots, \lambda_n), \quad V = [v_{ij}] \in \mathbb{R}^{n\times n}. \tag{15}$$

If we consider the spectral mapping analysis done in Section 2.2 we can reformulate $f(S)$ with more simple element-wise polynomial terms:

$$f(S) = \sum_{i=0}^{k} a_i (V\Lambda V^\top)^i = V\Big(\sum_{i=0}^{k} a_i \Lambda^i\Big)V^\top = V\Big(\sum_{i=0}^{k} a_i \mathrm{diag}(\lambda^i)\Big)V^\top. \tag{16}$$

2.4 Rotation Matrix

We cannot simply set $\hat{F} = f(S)$, as this would produce a matrix whose eigenvectors align with those of S. However, the eigenvectors of S and F generally span different eigenspaces. As a result, such a mapping would fail to capture the actual structure of F.

To illustrate this, we consider a toy example. In the figures 2 and 3 , we visualize the eigenspaces of toy S and F matrices to highlight their differences.

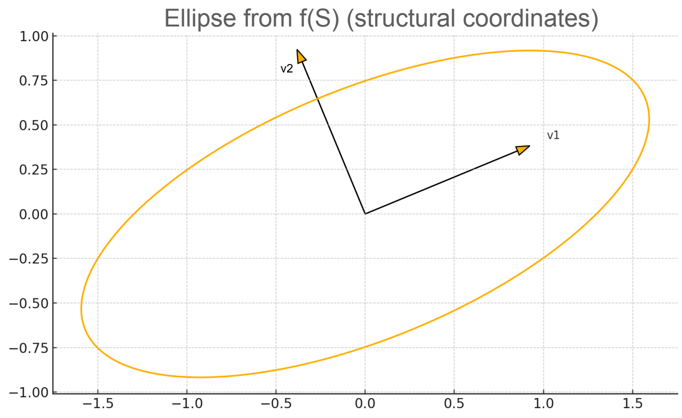

Figure 2. Illustration of the mapping $f(S) = a_0 I + a_1 S$ in the structural eigenspace. This figure shows the unit circle mapped through the toy structural matrix S using a first-order polynomial $f(S) = a_0 I + a_1 S$. The result is an ellipse (in orange) whose principal axes align with the eigenvectors $\mathbf{v}_1$ and $\mathbf{v}_2$ of S, scaled according to the polynomial coefficients. This transformation remains locked into the structural eigenbasis (V-basis), meaning that the directions of deformation are entirely governed by the structure of S, regardless of the target matrix F. This highlights the limitation of using $f(S)$ directly to approximate F: although the spectral values may be matched, the directional (eigenvector) information is misaligned.

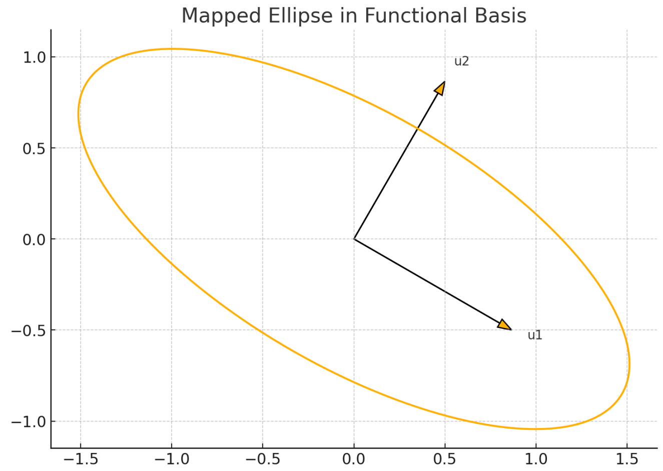

Figure 3. Illustration of the target functional matrix F in its eigenbasis. In this figure, the same unit circle is mapped through the toy functional connectivity matrix F, producing a new ellipse (in orange). The ellipse now aligns with the eigenvectors $\mathbf{u}_1$ and $\mathbf{u}_2$ of F, which define the functional eigenbasis (U-basis). Although the shape of the ellipse may be similar in scale to the one from $f(S)$, its orientation reflects the distinct eigenspace of F. This visual discrepancy demonstrates that directly using $f(S)$ without a change of basis cannot capture the correct geometry of F; both eigenvalues *and* eigenvectors must be accounted for, motivating the introduction of a rotation matrix $R \in \mathrm{SO}(n)$ to realign the eigenspaces.

This example highlights the core issue: without a change of basis, it is not possible to simultaneously match both the eigenvalues and eigenvectors of S and F.

If we select the coefficients a_i to ensure that the eigenvalues of $f(S)$ match those of F , the resulting matrix will still be misaligned in terms of direction, it will have incorrect eigenvectors. Conversely, if we force the eigenvectors to align, we lose the correct spectral magnitudes.

To overcome this, we must perform a change of eigen-basis to align the spectra correctly. Specifically, to match the empirical functional eigenspace, we introduce a rotation matrix $R \in \mathrm{SO}(n)$, resulting in

$$\hat{F}(S) = Rf(S)R^\top = RV\left(\sum_{i=0}^{k} a_i \mathrm{diag}(\lambda^i)\right)V^\top R^\top. \tag{17}$$

This R matrix needs to have some specific proprieties. It should represent a pure rotation, preserving the magnitude and distances in the transformed space without any scaling or distortion, representing a change of coordinates. For that, it is mandatory that the $det(R) = 1$ because the determinant of a matrix, in this case R, equals the volume of a box in n-dimensional space[3]. The edges of the box come from the rows of R, in Figure 4. The columns of R would give an entirely different box with the same volume.

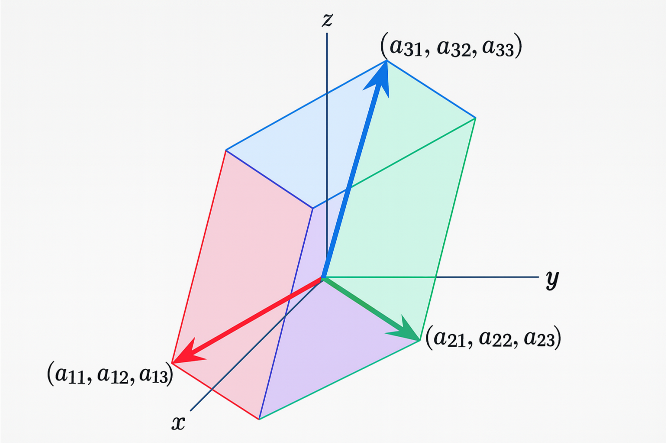

Figure 4. Geometric interpretation of the rotation matrix R in $\mathbb{R}^3$. The figure shows the parallelepiped formed by the row vectors of R, with each edge corresponding to one of the rows. The volume of this box equals the absolute value of $\det(R)$, which must be unitary to preserve orientation and volume under rotation. This ensures that $R \in \mathrm{SO}(n)$, is a proper rotation matrix with no reflection or scaling.

Knowing that the determinant should be unitary, in order to keep the eigen space and preserving the magnitude and distances, we can think that R should integrate the group of orthogonal matrices, which have their determinant unitary, as prooved below.

Proposition 1. *If $R \in \mathbb{R}^{n\times n}$ is orthogonal, i.e. $R^T R = I$, then* $\det(R) = \pm 1$. *Equivalently, an orthogonal matrix either preserves or reverses oriented volume.*

Proof. Since R is orthogonal,

$$R^T R = I.$$

Taking determinants and using $\det(AB) = \det(A)\det(B)$ and $\det(R^T) = \det(R)$ gives

$$\det(R^T R) \;=\; \det(I) \;\Longrightarrow\; \det(R^T)\det(R) \;=\; 1 \;\Longrightarrow\; \det(R)^2 = 1. \tag{18}$$

Hence

$$\det(R) = +1 \quad \text{or} \quad \det(R) = -1. \tag{19}$$

Thus, the following holds:

- If $\det(R) = +1$, R is *orientation-preserving* and scales every volume element by $+1$. Equivalently, R is a "pure" rotation.
- If $\det(R) = -1$, R is *orientation-reversing* (a rotation combined with a reflection), still preserving volume magnitude but flipping its sign.

□

If $\det(R) = -1$, then R includes a reflection and is not in $\mathrm{SO}(n)$. However, one verifies easily that $(-R)\,f(S)\,(-R)^T = R\,f(S)\,R^T$.
Since the two factors of -1 cancel. Moreover, $\det(-R) = (-1)^n \det(R) = (-1)^n(-1)$. In particular, when n is odd, $\det(-R) = +1$, and $-R$ is a valid pure rotation in $\mathrm{SO}(n)$ yielding the exact same mapped matrix $\hat{F}$.

3 Supplementary methods: Cumulative-contributions

Using the strategies described in Section (2), we seek to infer an individual functional connectivity matrix $F \in \mathbb{R}^{n\times n}$ from the corresponding structural connectivity matrix $S \in \mathbb{R}^{n\times n}$. This *structure–function mapping* problem is approached here through the spectral methodology introduced by Becker *et al.*[2]. Specifically, we approximate the unknown mapping by a finite-degree polynomial in S, an approach we call *individual spectral mapping*.

Let $k \in \mathbb{N}$ denote the maximum walk length considered. The model can take the form as seen before

$$f(S) \;=\; \sum_{i=0}^{k} a_i S^i, \tag{20}$$

where the coefficients $a_i \in \mathbb{R}$ $(i = 0,\dots,k)$ are estimated from data. The identity term $S^0 \equiv I_n$ captures intrinsic (zero-step) interactions, while the higher powers S^i $(i \geq 1)$ encode walks of length i in the structural network. Appropriate weighting by $\{a_i\}$ therefore enables the model to balance direct and polysynaptic pathways, yielding a concise yet flexible representation of the structure–function relationship.

Because (20) is a *sum* of successive powers, each additional term augments the information already supplied by shorter walks. Consequently, $f(S)$ integrates the *cumulative* contribution of *all* walks whose length does not exceed k. Increasing k, therefore, broadens the dynamic range by progressively bringing longer anatomical pathways into play, while still retaining the weights of all shorter walks.

Considering that S is symmetric, we use its eigen-decomposition as in (14)–(15). Applying this decomposition to (20) yields

$$f(S) = \sum_{i=0}^{k} a_i \left(V\Lambda V^\top\right)^i = \sum_{i=0}^{k} a_i V \Lambda^i V^\top = V\Big(\sum_{i=0}^{k} a_i \operatorname{diag}(\lambda^i)\Big)V^\top, \tag{21}$$

where $\operatorname{diag}(\lambda^i)$ denotes the diagonal matrix of ith powers of λ.

Introducing $R \in \mathrm{SO}(n)$, the rotated prediction becomes

$$\hat{F}(S) = Rf(S)R^\top = RV\Big(\sum_{i=0}^{k} a_i \operatorname{diag}(\lambda^i)\Big)V^\top R^\top. \tag{22}$$

The unknown rotation R and coefficients $\{a_i\}$ are then obtained by minimising the Frobenius-norm misfit:

$$\begin{aligned} \min_{R,\{a_i\}} \quad & \|F - \hat{F}(S)\|_F^2 = \sum_{i,j}([\hat{F}]_{ij} - [F]_{ij})^2, \\ \text{s.t.} \quad & RR^\top = R^\top R = I_n. \end{aligned} \tag{23}$$

The optimization task can than be written from (23) and (21) in to

$$\min_{a_0,\dots,a_k,R} \left\|F - \hat{F}\right\|_F^2 = \min_{a_0,\dots,a_k,R} \left\| F - RV\Big(\sum_{i=0}^{k} a_i \operatorname{diag}(\lambda^i)\Big)V^\top R^\top \right\|_F^2 \quad \text{subject to } R^\top R = I. \tag{24}$$

3.1 Finding initial values for optimization - Warm-start

Following Becker *et al.*[2], we initialize at

$$R^\star = UV^\top, \quad \operatorname{diag}(\varphi) \approx \sum_{i=0}^{k} a_i \operatorname{diag}(\lambda^i), \tag{25}$$

which drives the norm in (23) to zero for k to ∞. For finite k, we solve

$$\min_{\{a_i\}} \left\|\operatorname{diag}(\varphi) - \sum_{i=0}^{k} a_i \operatorname{diag}(\lambda^i)\right\|_F^2, \tag{26}$$

via the Vandermonde matrix

$$L = \begin{bmatrix} 1 & \lambda_1 & \cdots & \lambda_1^k \\ \vdots & & \ddots & \\ 1 & \lambda_n & \cdots & \lambda_n^k \end{bmatrix}, \quad a^* = (L^T L)^{-1} L^T \varphi. \tag{27}$$

3.2 Optimisation Process

After finding the initial values for optimization, we can advance to the optimization and iterative phase. Equation (24) constrains the rotation matrix $R \in \mathrm{SO}(n) = \{Q \in \mathbb{R}^{n\times n} \mid Q^\top Q = I$, a smooth[1] but curved manifold. Standard unconstrained algorithms operate in the Euclidean space $\mathbb{R}^{n\times n}$ and would therefore leave $\mathrm{SO}(n)$. To solve this issue, we adopt a *Riemannian-optimisation* framework.

In the proposed framework we start the optimization process by defining a mixed search space, given by:

$$\mathscr{M} \;=\; \underbrace{\mathrm{SO}(n)}_{R} \times \underbrace{\mathbb{R}^{k+1}}_{\mathbf{a}}, \qquad x = (R, \mathbf{a}) \in \mathscr{M}, \quad \mathbf{a} = (a_0, \ldots, a_k)^\top. \tag{28}$$

Where R lives in a non-euclidian space and the coefficients $a = (a_0, \ldots, a_k)^\top$, live on an euclidean space.

The cost function

$$f \,:\, \mathscr{M} \longrightarrow \mathbb{R}, \qquad f(R, \mathbf{a}) = \left\| F - RV\big(\textstyle\sum_{\ell=0}^{k} a_\ell diag(\lambda^\ell)\big)V^\top R^\top \right\|_F^2, \tag{29}$$

depends on $V \in \mathbb{R}^{n\times n}$ (the eigenvectors of S) and $\lambda \in \mathbb{R}^n$ (the associated eigenvalues).

With respect to the gradients, at iteration t we compute the *Euclidean* derivatives $\nabla_R f \in \mathbb{R}^{n\times n}$ and $\nabla_{\mathbf{a}} f \in \mathbb{R}^{k+1}$. Because R is constrained to $\mathrm{SO}(n)$, only directions inside its tangent space are admissible.

Because R is required to remain in the special-orthogonal group, a legitimate search direction must belong to the tangent space.

To obtain such a direction we orthogonally project the full matrix $\nabla_R f$ onto this tangent space. The projection operator is denoted $\Pi_R : \mathbb{R}^{n\times n} \to \mathscr{T}_R \mathrm{SO}(n)$:

$$\mathrm{grad}_R f \;=\; \Pi_R\big(\nabla_R f\big) \tag{30}$$

Here, $\nabla_R f$ is the *Euclidean* gradient with respect to R, and the resulting matrix $\mathrm{grad}_R f$ is the Riemannian gradient of f at R.

For the polynomial coefficients $\mathbf{a} = (a_0, \ldots, a_k)^\top \in \mathbb{R}^{k+1}$, which are not subject to any manifold constraint, the Riemannian and Euclidean gradients coincide:

$$\mathrm{grad}_{\mathbf{a}} f \;=\; \nabla_{\mathbf{a}} f \;\in\; \mathbb{R}^{k+1}. \tag{31}$$

These two quantities, $\mathrm{grad}_R f$ and $\mathrm{grad}_{\mathbf{a}} f$, together provide the full gradient on the product manifold $\mathscr{M} = \mathrm{SO}(n) \times \mathbb{R}^{k+1}$ used by the Riemannian optimisation algorithm.

Considering the search direction and retraction part, if we let $\Delta_t = (\Delta_R, \Delta_{\mathbf{a}}) \in \mathscr{T}_{R_t}\mathrm{SO}(n) \times \mathbb{R}^{k+1}$ be a search direction. A step of length $\eta_t > 0$ produces the tentative update

$$\widetilde{R} = R_t + \eta_t \Delta_R, \qquad \widetilde{\mathbf{a}} = \mathbf{a}_t - \eta_t \Delta_{\mathbf{a}}. \tag{32}$$

Because the tentative update $\widetilde{R} = R_t + \eta_t \Delta_R$ does not necessarily satisfy the orthogonality constraint, we map it back to the manifold with a retraction.

$$R_{t+1} = \mathrm{Retr}_{R_t}\big(\eta_t \Delta_R\big), \qquad \mathbf{a}_{t+1} = \mathbf{a}_t - \eta_t \Delta_{\mathbf{a}}. \tag{33}$$

The operator $\mathrm{Retr}_R(\cdot)$ guarantees that $R_{t+1} \in \mathrm{SO}(n)$ while preserving the local geometry, so the new pair $(R_{t+1}, \mathbf{a}_{t+1})$ again lies in the product manifold $\mathscr{M} = \mathrm{SO}(n) \times \mathbb{R}^{k+1}$ and serves as the next iterate. The concrete numerical realisation of this retraction is detailed in the implementation details subsection (3.3).

[1] $\mathrm{SO}(n)$ is a compact Lie group of dimension $n(n-1)/2$.

3.3 Implementation details (MANOPT baseline)

All optimisation routines were implemented in MATLAB using the MANOPT toolbox [4]. The optimization process was done following the steps indicated below:

- **Rotation component.** The factor `rotationsfactory(n)` initiates the Riemannian geometry of the special-orthogonal group $\mathrm{SO}(n) = \{R \in \mathbb{R}^{n\times n} \mid R^\top R = I, \det R = 1\}$. It provides intrinsic routines for projection (`proj`), retraction (`retr`), and conversion from Euclidean to Riemannian gradients (`egrad2rgrad`).
- **Coefficient component.** The vector of polynomial coefficients $\mathbf{a} = (a_0, \ldots, a_k)^\top \in \mathbb{R}^{k+1}$ is modelled by the Euclidean manifold `euclideanfactory(k+1,1)`.
- **Cartesian product.** The full search space $\mathscr{M} = \mathrm{SO}(n) \times \mathbb{R}^{k+1}$ is declared via `productmanifold` with the two components above.

To calculate the costs and gradients, we proceed in the following way:

- **Cost handle.** The objective in (24) is coded in `frob_error_combined.m`. Given a point $x = (R, \mathbf{a})$, it builds the spectral filter in the structural eigenbasis, forms $\hat{F}$, and returns $\|F - \hat{F}\|_F^2$.
- **Euclidean gradient.** Closed-form derivatives with respect to R and $\mathbf{a}$ are implemented in `frob_grad_combined.m`. manopt automatically projects $\nabla_R f$ onto $\mathscr{T}_R\mathrm{SO}(n)$ and leaves $\nabla_{\mathbf{a}} f$ unchanged.

The optimisation is launched with the Riemannian L-BFGS routine `rlbfgs`. In our experiments Riemannian L-BFGS with step size 0.1 and tolerance 10^{-8} yielded a reasonable trade-off between speed and robustness. Preliminary experiments confirmed that these hyper-parameters balance speed and robustness across all subjects and walk lengths $k \leq 10$. For comparison, the conjugate-gradient and trust-region solvers yielded similar solutions but required more computation time.

Stopping criterion. Iterations terminate when $\|\operatorname{grad} f(x_t)\|_2 < 10^{-8}$ or when the step size drops below 10^{-12}. In practice the gradient tolerance is met first.

3.4 Convexity

There are great advantages to recognizing a problem as a convex optimization problem. The most basic advantage is that the problem can then be solved, very reliably and efficiently, using interior-point methods or other special methods for convex optimization[5]. To reach that goal, we must characterise the landscape of the objective function $f(R,a)$, describe by the optimization problem represented by (24). To characterise the optimisation landscape we begin by recalling the notion of convexity in Euclidean space[5]. A function $g : \mathbb{R}^d \to \mathbb{R}$ is convex if, for any two points $x, y \in \mathbb{R}^d$ and any $t \in [0,1]$,

$$g\big(t\,x + (1-t)\,y\big) \;\leq\; t\,g(x) \;+\; (1-t)\,g(y). \tag{34}$$

Equivalently, its Hessian is positive semidefinite everywhere, a synonym of convexity [5], which guarantees that all local minima are global minima, a characteristic that simplifies the optimization process if verified.

In our problem, however, the orthogonality constraint $R^T R = I$ forces the rotation variable R to lie on the manifold $\mathrm{SO}(n)$ rather than in a Euclidean space. This non-Euclidean geometry invalidates the standard euclidean notion of convexity, since it cannot form arbitrary linear interpolants between two rotations.

To generalise convexity to this setting, one replaces the straight segment $t\,x + (1-t)\,y$ by the geodesic $\gamma(t)$ on the Riemannian product manifold $\mathrm{SO}(n) \times \mathbb{R}^{k+1}$. A function f on this manifold is said to be geodesically convex if, for every pair of feasible points (R_1, a_1) and (R_2, a_2), and for every $t \in [0,1]$,

$$f\big(\gamma(t)\big) \;\leq\; t\,f(R_1, a_1) \;+\; (1-t)\,f(R_2, a_2), \tag{35}$$

where $\gamma(0) = (R_2, a_2)$, $\gamma(1) = (R_1, a_1)$, and $\gamma(t)$ traverses the shortest path in $\mathrm{SO}(n) \times \mathbb{R}^{k+1}$. Geodesic convexity is equivalently characterised by positive semidefiniteness of the Riemannian Hessian on each tangent space, and likewise ensures that any local minimum is global.

By simplifyng the problem and constructing a two-dimensional toy case in $\mathrm{SO}(2) \times \mathbb{R}$, we can verify this Jensen inequality.

For that, if we consider $k = 1$, $a_0 = 0$ and $a_1 = a \in \Re$, we force $f(S) = aS$ and the modified optimization function is given by

$$\min_{R,a_0,a_1} \left\| U\, diag(\varphi)\, U^\top - R V\, diag\big(a_0 + a_1 \lambda_i^k\big)\, V^\top R^\top \right\|_F^2 \quad \text{subject to } R^\top R = I. \tag{36}$$

This modified problem, implies that ($R \in \mathrm{SO}(2)$), so a possible R matrix that complies with the orthogonal restriction could be

$$R = \begin{bmatrix} \cos\theta & -\sin\theta \\ \sin\theta & \cos\theta \end{bmatrix}, \tag{37}$$

which represents a valid rotation by angle θ and satisfies $R(\theta)^\top R(\theta) = I$.

A convenient way to assess the convexity of the objective is to visualise its simplified form through contour and surface plots, that are presented in Figures 5a and 5b.

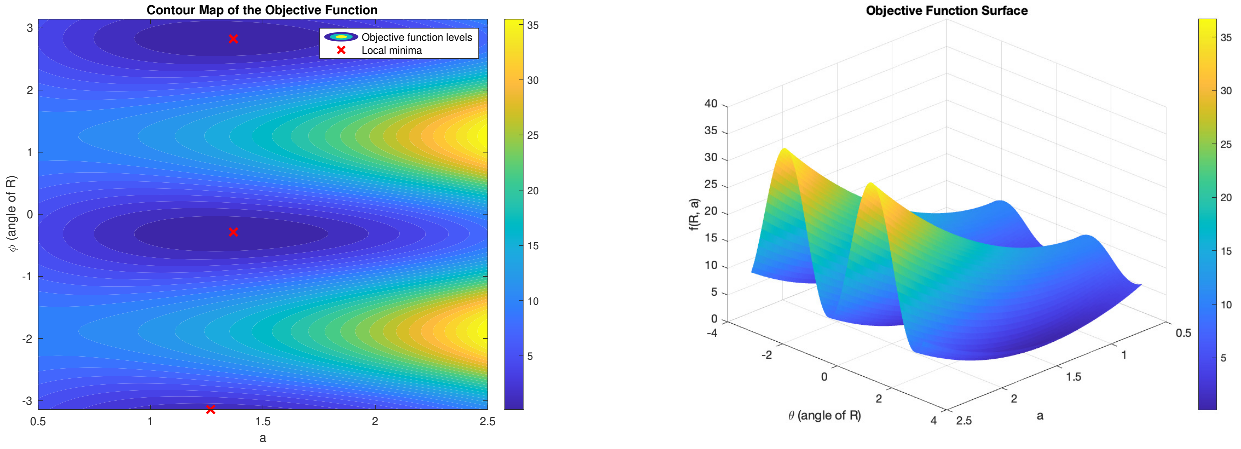

(a) Contour (2-D) view

(b) Surface (3-D) view

Figure 5. Side-by-side visualisation of the simplified objective function: (a) contour plot and (b) surface plot. Together they reveal the presence of multiple valleys, confirming the non-convex character of the objective function.

After visualizing non-convexity, we also prove that the function violates Jensen's inequality, establishing non-convexity. To test the inequality in (36) on this manifold we pick two feasible points

$$(R_1, a_1) = (R(0), 0.8), \qquad (R_2, a_2) = (R(\pi), 2.5), \tag{38}$$

where $R(\theta)$ is the rotation matrix defined above. The unique geodesic $\gamma : [0,1] \to \mathrm{SO}(2) \times \mathbb{R}$ joining these points is given by

$$\gamma(t) = \big(R(t\pi),\, (1-t)a_1 + t\, a_2\big), \qquad t \in [0,1], \tag{39}$$

so that the midpoint is $\gamma(1/2) = (R(\pi/2), 1.65)$. Evaluating the objective we obtain

$$f(R_1, a_1) = 2.9592, \quad f(R_2, a_2) = 10.9900, \quad f\big(\gamma(\tfrac{1}{2})\big) = 17.6173. \tag{40}$$

Jensen's geodesic condition would require $f\big(\gamma(\frac{1}{2})\big) \le \frac{1}{2} f(R_1, a_1) + \frac{1}{2} f(R_2, a_2) = 6.9746$, but the computed values violate this inequality $17.6173 > 6.9746$.

Hence, even in the two–dimensional setting the objective is *not* geodesically convex, implying that the full high–dimensional problem inherits a non-convex landscape with multiple local minima.

4 Supplementary methods: Individual–contributions

Where the cumulative model in Section 3 sums every walk up to order k (20), here we isolate the predictive power of a *single* walk length $k \in \{1,\dots,k_{\max}\}$. The structural-function mapping therefore reduces to the smaller parameter model

$$f_k(S) \;=\; a_0 I_n \;+\; a_k S^k, \qquad a_0, a_k \in \mathbb{R}, \tag{41}$$

which is fitted independently for each k. This restriction removes all dependences from walks sizes lower than k that characterise the cumulative approach.

4.1 Spectral least–squares fit on eigenvalues

Let $S = V\,\mathrm{diag}(\lambda)\,V^\top$ be the eigendecomposition of the structural matrix with eigenvalues $\lambda = (\lambda_1,\dots,\lambda_n)^\top$. Substituting into (41) yields the spectral form

$$f_k(S) = V\Big(a_0 I + a_k \lambda^k\Big)V^\top, \tag{42}$$

with λ^k denoting the element–wise k-th power. Analogous to (27) in the cumulative section, the Vandermonde-type matrix for the individual fit is

$$L_k = \begin{bmatrix} 1 & \lambda_1^k \\ \vdots & \vdots \\ 1 & \lambda_n^k \end{bmatrix}, \qquad \varphi = (\varphi_1,\dots,\varphi_n)^\top, \tag{43}$$

where φ_i are the eigenvalues of the empirical functional matrix in its own basis. Solving the 2×2 normal equations gives

$$\begin{bmatrix} a_0^* \\ a_k^* \end{bmatrix} = (L_k^\top L_k)^{-1} L_k^\top \varphi. \tag{44}$$

In contrast to the $(k_{\max}+1)$-column system faced in the cumulative model, (42) contains only two columns, minimizing complexity.

4.2 Rotation step and optimisation problem

Reintroducing orientation, the functional prediction associated with walk length k becomes

$$\widehat{F}_k = R\,f_k(S)\,R^\top = RV\Big(a_0 I + a_k \lambda^k\Big)V^\top R^\top, \tag{45}$$

so that the unknowns (a_0, a_k, R) are obtained from the Frobenius–norm mis-fit

$$\min_{R, a_0, a_k} \; \|F - RV(a_0 I_n + a_k S^k)V^\top R^\top\|_F^2, \quad \text{s.t. } R^T R = RR^T = I_n. \tag{46}$$

which lives on the product manifold $\mathscr{M} = \mathrm{SO}(n) \times \mathbb{R}^2$ ((28) for the cumulative analogue).

4.3 Finding initial values for optimisation — Warm-start

The warm-start follows exactly the procedure used in the cumulative case (see (25)). First, we align the eigen-bases of the structural and functional matrices by setting $R^* = UV^\top$, where U contains the eigenvectors of F. With this rotation held fixed, we then obtain the coefficient estimates a_0^* and a_k^* from (44). optimisation.

4.4 Optimisation process

As in Section 3.2, we adopt a mixed Riemannian framework: R lives on $\mathrm{SO}(n)$ while (a_0, a_k) lie in an unconstrained Euclidean space. Manopt factories and dimensions are summarised below:

Component	Manopt factory	Dimension
Rotation R	`rotationsfactory(n)`	$n(n-1)/2$
Coeffs. (a_0, a_k)	`euclideanfactory(2,1)`	2

The cost and gradient handles are identical to the cumulative approach except that the spectral model that now uses (46). We launch `rlbfgs` with step size 0.1 and tolerance 10^{-8}; stopping criteria mirror those in the cumulative section 3. The convexity analysis presented in Section 3.4 applies unchanged to the individual–contributions model.

5 Spatial Alignment with the Network Correspondence Toolbox

To determine what regions are involved in the results obtained in the main article, we quantified the spatial correspondence between individual hub maps and multiple canonical atlases. Demonstrating a preferential alignment (or mis-alignment) with specific networks can provide insights into disease-related reorganisation of the connectome and can, in this case, help pinpoint which regions may mediate migraine pathology.

The spatial overlap between our subject–level hub maps and canonical functional atlases was quantified with the Network Correspondence Toolbox (NCT),[6]. which first projects each volumetric map, then computes Dice coefficients, and finally assesses significance with spatial "spin" permutations that preserve surface autocorrelation.

For each group (patients and controls), the computation pipeline was the following:

1. The functional connectivity (FC) matrix $F \in [-1,1]^{130\times130}$ was summed row by row to obtain a degree vector. $d_i = \sum_j F_{ij}$.
2. Voxels belonging to ROIs whose degree exceeded the 75th percentile (top 25%) were assigned the value 1; others 0. This yielded a binary individual hub mask H_{subj}.
3. For every reference network A_{ref} the Dice coefficient $\text{Dice}(H_{\text{subj}}, A_{\text{ref}}) = \frac{2|H_{\text{subj}} \cap A_{\text{ref}}|}{|H_{\text{subj}}|+|A_{\text{ref}}|}$ was computed.
4. Spatial significance was evaluated with $N_{\text{perm}} = 5{,}000$ random rotations (*spin test*)[7]. An empirical p-value was assigned as the proportion of spins whose Dice $\geq$ the observed value.
5. NCT's "network-clock" and "radar" plots were generated for every group and are displayed in Fig. 6 and 7 on the main article, and in the Tables 2 and 3.

The complete workflow of the Network Correspondence Toolbox and an example of its summary outputs are illustrated in Figure 6.

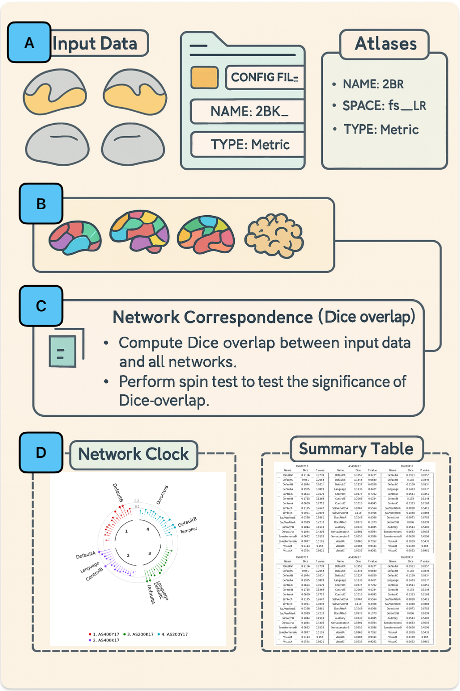

Figure 6. Visual workflow and summary outputs of the network correspondence analysis. This figure summarizes the use of the CBIG Network Correspondence Toolbox (NCT) to evaluate the alignment between functional hub maps and canonical brain networks. **(A)** Subject-specific input data, including cortical hub maps and configuration files, are defined alongside the target atlas list. **(B)** Selected network atlases are aligned to the subject space for comparison. **(C)** The core analysis computes Dice overlap between hub maps and reference networks, and performs permutation-based spin tests to assess statistical significance. **(D)** Final outputs include a *Network Clock* plot showing Dice values across networks, and a *Summary Table* reporting Dice coefficients and empirical p-values.

6 Correlation Metrics

To quantify how well the predicted functional connectivity matrix $\hat{F}$ approximates the empirical matrix F, we evaluated three complementary metrics across walk lengths $k \in \{1, \dots, 7\}$. Each metric captures a different aspect of approximation quality and is visualized for both control and patient groups in Figures 7 and 8.

1. The Frobenius error quantifies the total squared difference between F and $\hat{F}$:

$$E_{\text{Frob}}(k) = \|F - \hat{F}\|_F^2 = \sum_{i=1}^{n} \sum_{j=1}^{n} \left(F_{ij} - \hat{F}_{ij}\right)^2.$$

   It reflects the total prediction error, including both structure and scale. In both groups, this metric drops sharply for small k and plateaus beyond $k = 3$ (in blue, in Figures 7 and 8).

2. The upper-triangular (off-diagonal) Pearson correlation assesses similarity in connection patterns, independent of absolute scale. It is defined as:

$$\rho_{\text{off}}(k) = \text{corr}(x, y) = \frac{\sum_m (x_m - \bar{x})(y_m - \bar{y})}{\sqrt{\sum_m (x_m - \bar{x})^2} \sqrt{\sum_m (y_m - \bar{y})^2}},$$

   where x and y are the vectorized upper triangles of F and $\hat{F}$. This is sensitive to pattern alignment but invariant to overall magnitude. This correlation increases rapidly up to $k = 2$ and decreases slightly beyond that point (in green, in Figures 7 and 8).

3. The spectral-domain Frobenius error measures how well the predicted eigenvalues match the true spectrum of F in the aligned basis. With λ_j the eigenvalues of S and φ_j the aligned eigenvalues of F, we compute

$$E_{\text{specVec}}(k) = \|\hat{\varphi} - \varphi\|_2^2 = \sum_{j=1}^{n} \left(a_0 + a_1 \lambda_j^k - \varphi_j\right)^2.$$

   This metric isolates spectral approximation error and is particularly informative when analysing models constrained to fixed walk lengths. It is shown in red on Figures 7 (controls) and 8 (patients).

These three metrics provide an understanding perspective of the model performance. They are used throughout the study to evaluate reconstruction quality, compare models, and to analyse robustness across cohorts.

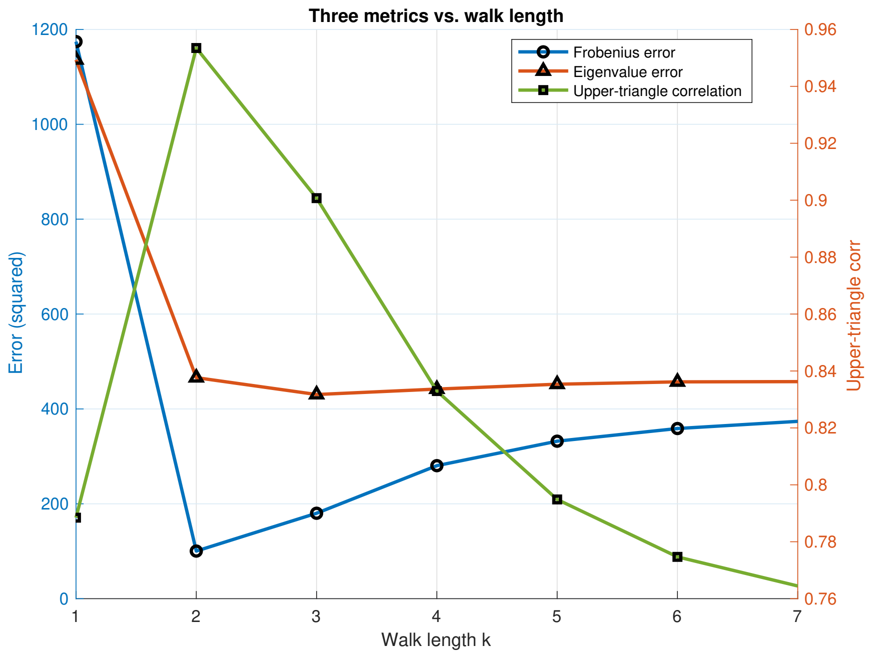

Figure 7. Control group: summary of three error metrics. (Blue) Frobenius error quantifies residual energy between predicted and empirical connectivity. (Green)) Upper-triangle Pearson correlation measures pattern alignment. (Red) Spectral-domain error measures eigenvalue misfit in the aligned eigenspace of F.

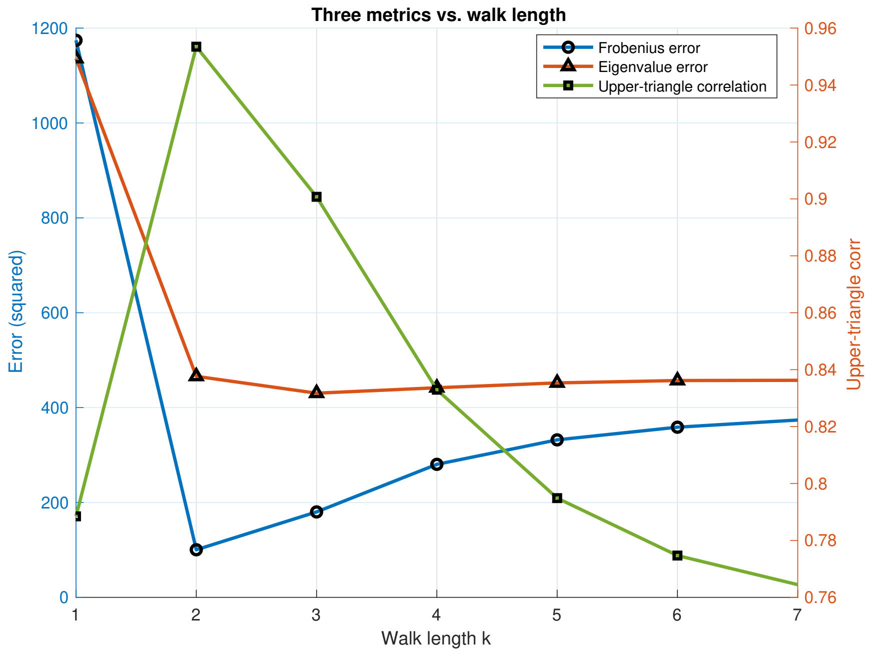

Figure 8. Patient group: summary of three error metrics. Same layout as Figure 7, showing error trends and correlation profiles for the migraine group across walk lengths $k = 1, \ldots, 7$.

7 Perturbation Analysis

The process of obtaining the experimental matrices S and F for each individual is exposed to noise. The goal is then to test how well the spectral mapping between structural S and functional F connectivity matrices holds up when noise is introduced to the structural connectivity matrix S, helping to understand if the mapping process is reliable under realistic imperfections in data.

To that propose, let's consider simple and examplificative 4×4 structural S and functional F connectivity matrices. Let's consider, two randomly generated matrix that maintain the main characteristics of the matrices S and F.

$$F = \begin{bmatrix} 1.0000 & 0.4119 & 0.6857 & 0.6314 \\ 0.4119 & 1.0000 & 0.8290 & 0.5109 \\ 0.6857 & 0.8290 & 1.0000 & 0.3835 \\ 0.6314 & 0.5109 & 0.3835 & 1.0000 \end{bmatrix} \quad S = \begin{bmatrix} 1.0000 & 0.4763 & 0.4594 & 0.7309 \\ 0.4763 & 1.0000 & 0.1207 & 0.6043 \\ 0.4594 & 0.1207 & 1.0000 & 0.5647 \\ 0.7309 & 0.6043 & 0.5647 & 1.0000 \end{bmatrix}$$

With these matrices, it is possible, by solving the optimization problem described in (24), to obtain the coefficients $\{a_i\}_{i=0}^{k}$ and the rotation matrix R, which allow the mapping to be performed.

To evaluate the robustness of the mapping, a new perturbed structural connectivity matrix, denoted by $S^{\vee}$, is computed using a random multiplicative perturbation model.

For each element $[S]_{ij}$ of the structural connectivity (SC) matrix, a random value $[\Delta]_{ij}$ is sampled from a uniform distribution between $-\rho$ and ρ, where ρ is the maximum magnitude of the perturbation. The perturbed value is then computed as

$$[S^{\vee}]_{ij} = (1 + [\Delta]_{ij})\, [S]_{ij}, \tag{47}$$

where $S^{\vee}$ denotes the perturbed structural matrix.

This model ensures that the perturbation magnitude is proportional to the original value of each connection. Stronger connections are perturbed more than weaker ones, reflecting realistic variations in data.

The perturbed matrix $S^{\vee}$, the coefficients $\{a_i\}_{i=0}^{k}$, and the rotation matrix R are then used in (24) to predict the functional connectivity matrix $F^{\vee}$.

To assess robustness, the original functional connectivity (FC) matrix F and the perturbed matrix $F^{\vee}$ are compared using a correlation measure:

$$u_{\text{corr}}(F, F^{\vee}), \tag{48}$$

where u_{corr} denotes the correlation measure.

A high correlation value indicates that the mapping is robust to perturbations, as the perturbed matrix $F^{\vee}$ remains similar to the original matrix F.

The robustness is analysed for different values of the perturbation parameter ρ, which controls the noise level. The results allow to understand how sensitive the spectral mapping is to variations in the structural data and are presented in Figure 9.

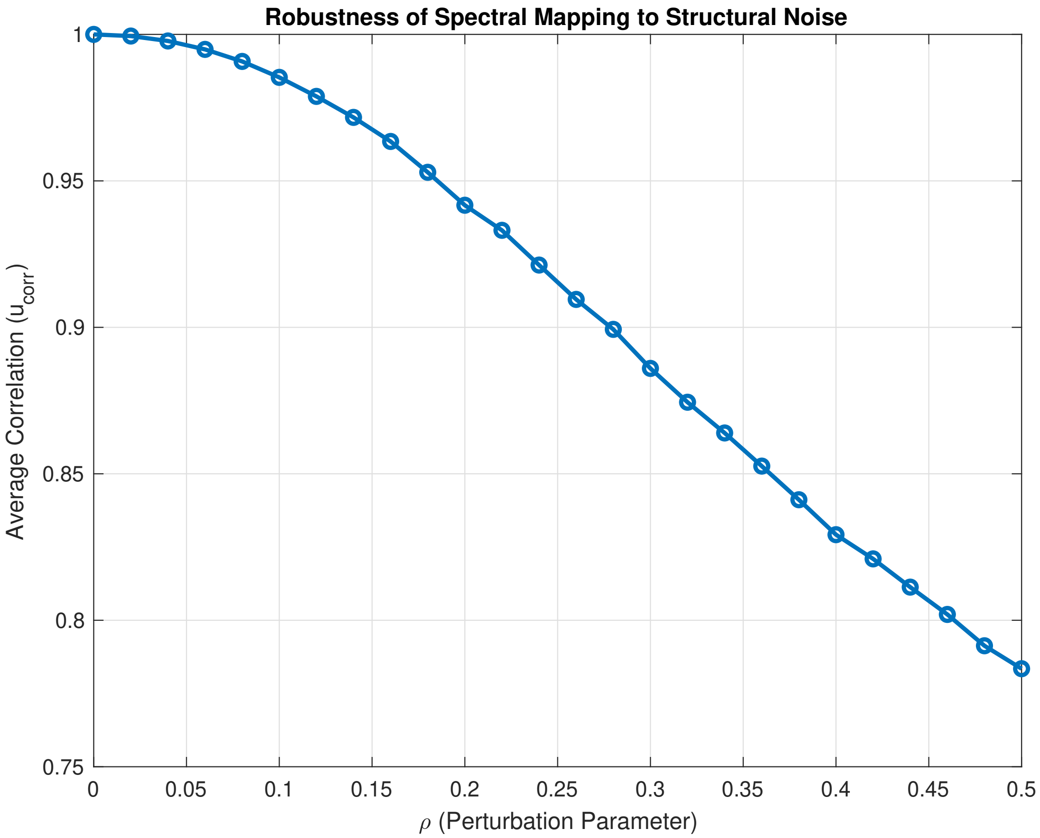

Figure 9. Impact of perturbation parameter ρ on the correlation measure u_{corr}.

In Figure 9 is illustrated the robustness of spectral mapping, obtained by considering walks of length less than or equal to 7. This choice was motivated by the observation that the median mapping quality saturates for $k \geq 8$, as reported by Becker et al.[2]. The plot represents the average correlation values (u_{corr}) across 10,000 independent runs, mitigating the influence of outliers caused by random noise. This averaging process ensures a reliable estimate of the mapping's robustness against perturbations.

The results indicate that spectral mapping remains highly robust under small perturbations, especially when the entries of the structural connectivity matrix are perturbed by at most 10% ($\rho \leq 0.1$). However, for $\rho > 0.1$, the correlation quality begins to decline, following an almost linear trend with increasing perturbations.

As the structural matrices deviate more significantly from their original form, the method becomes less effective. Nevertheless, these findings highlight that the proposed mapping demonstrates considerable robustness with respect to bounded perturbations in the structural matrix.

Finally, when there is no noise, $\rho = 0$, the methodology proposed allows to fully recover ($u_{\text{corr}} = 1$) the functional connectivity matrix F, assuring the good formulation of the mapping.

8 Supplementary Results

Cumulative contributions of walk lengths in function matrix estimation

We fit the cumulative spectral mapping model (24) to characterize how indirect structural walks of increasing length contribute to the prediction of the empirical functional connectivity matrix $F \in \mathbb{R}^{130\times130}$.

Figure 10 summarizes the distribution of off-diagonal Pearson correlations between predicted and observed connectivity for walk lengths $k = 1, 2, \dots, 10$.

When we consider only direct structural connections ($k = 1$), the median correlation reaches 0.8305 in the control group and 0.7922 in the migraine group. Incorporating two-step walks ($k = 2$) increases the median correlations to 0.9851 (controls) and 0.9914 (patients), capturing over 95% of the final structure–function alignment. Adding three-step walks ($k = 3$) yields modest improvements: 0.9927 for controls and 0.9940 for patients. Further contributions diminish for $k \geq 4$ (e.g., $k = 4$: 0.9952 and 0.9963; $k = 5$: 0.9969 and 0.9974), and for $k \geq 8$ both groups exceed 0.9992. At $k = 10$, both cohorts approach perfect alignment (controls: 0.9996, patients: 0.9995).

We assess statistical differences between groups using two-sample Kolmogorov–Smirnov tests at each walk length. The null hypothesis asserts that the off-diagonal correlation distributions are identical between groups. At $k = 1$, $p = 0.062$, so we do not reject the null hypothesis at a significance level of $\alpha = 0.05$. For $2 \leq k \leq 10$, $p \gg 0.05$, indicating no significant differences once polysynaptic paths of length two or greater are included.

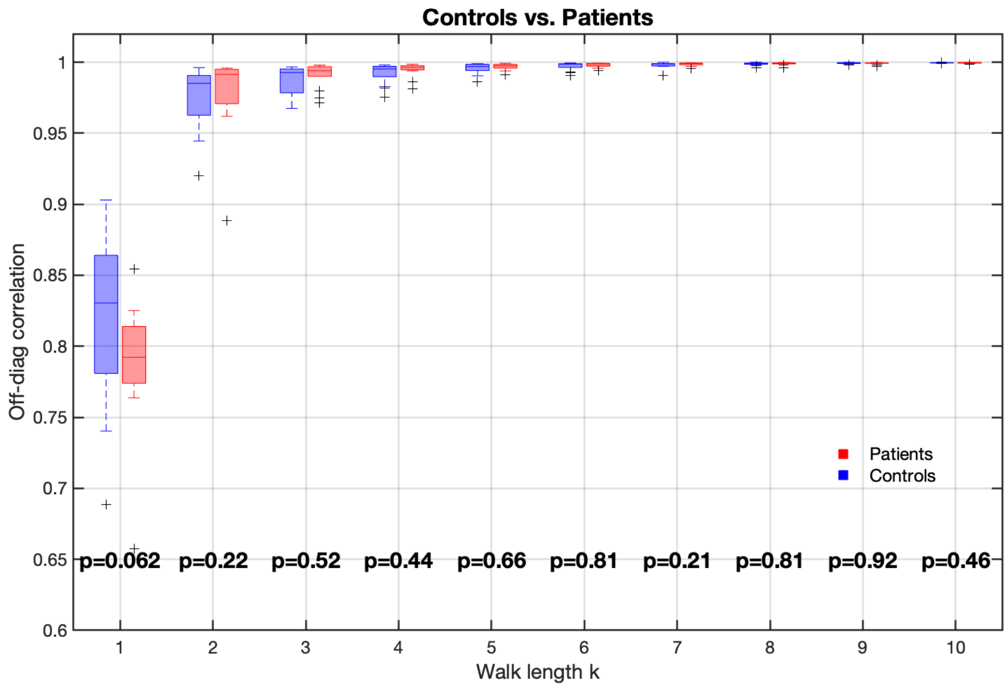

Figure 10. Boxplots of off-diagonal Pearson correlations between predicted and observed functional connectivity based on cumulative walk contributions for $k = 1:10$. Blue boxes denote controls ($n = 15$) and red boxes denote migraine patients ($n = 14$). The central mark indicates the median; box edges represent interquartile range; whiskers extend to $1.5 \times$ IQR; and crosses mark outliers. Two-sample Kolmogorov–Smirnov p-values appear below each pair of boxplots.

These findings confirm that, aside from a non-significant deviation at $k = 1$, the cumulative effect of indirect structural walks on functional connectivity is preserved across both populations.

We quantify inter-subject variability via the interquartile range (IQR) of off-diagonal Pearson correlations at each walk length $k \in \mathbb{N}$.

At $k = 1$, the control group exhibits IQR $= 0.0831$, indicating substantial heterogeneity in monosynaptic predictive accuracy. The migraine group shows a narrower IQR $= 0.0400$, suggesting more uniform but overall attenuated first-order coupling.

At $k = 2$, both groups show a marked reduction in variability, with IQR $= 0.0280$ for controls and IQR $= 0.0242$ for patients, indicating that two-step polysynaptic pathways yield consistently high predictive accuracy across individuals.

As k increases further, IQRs continue to decrease: at $k = 3$, controls have IQR $= 0.0168$ and patients have IQR $= 0.0067$, for $k \geq 5$, both groups drop below 0.005, and at $k = 10$, controls reach IQR $= 0.0002$ and patients reach IQR $= 0.0005$, indicating near-ceiling correlations with negligible dispersion.

These results demonstrate that indirect structural walks of length greater than one not only improve predictive accuracy but also homogenize the structure–function mapping across individuals and groups.

Our observations align with the principle proposed by Becker et al.[2]: structural walks up to length three suffice to reconstruct large-scale functional networks with near-perfect fidelity, and mapping accuracy saturates for $k > 7$.

8.1 Bootstrapping

8.1.1 Two sided Bootstrap

To validate the group differences found, we applied a two-sided Kolmogorov–Smirnov (KS) under variability in the data across 10 non-parametric bootstrap resamples per subject. This approach assesses whether differences persist across surrogate datasets generated from the BOLD signal.

Figure 11 presents the resulting distribution of p-values across bootstraps for each walk length $k = 1, \ldots, 10$. Boxplots summarize the variability in significance, and a red dashed line indicates the $\alpha = 0.05$ threshold. This analysis allows us to identify walk lengths where the observed effect between groups remained robust under repeated sampling.

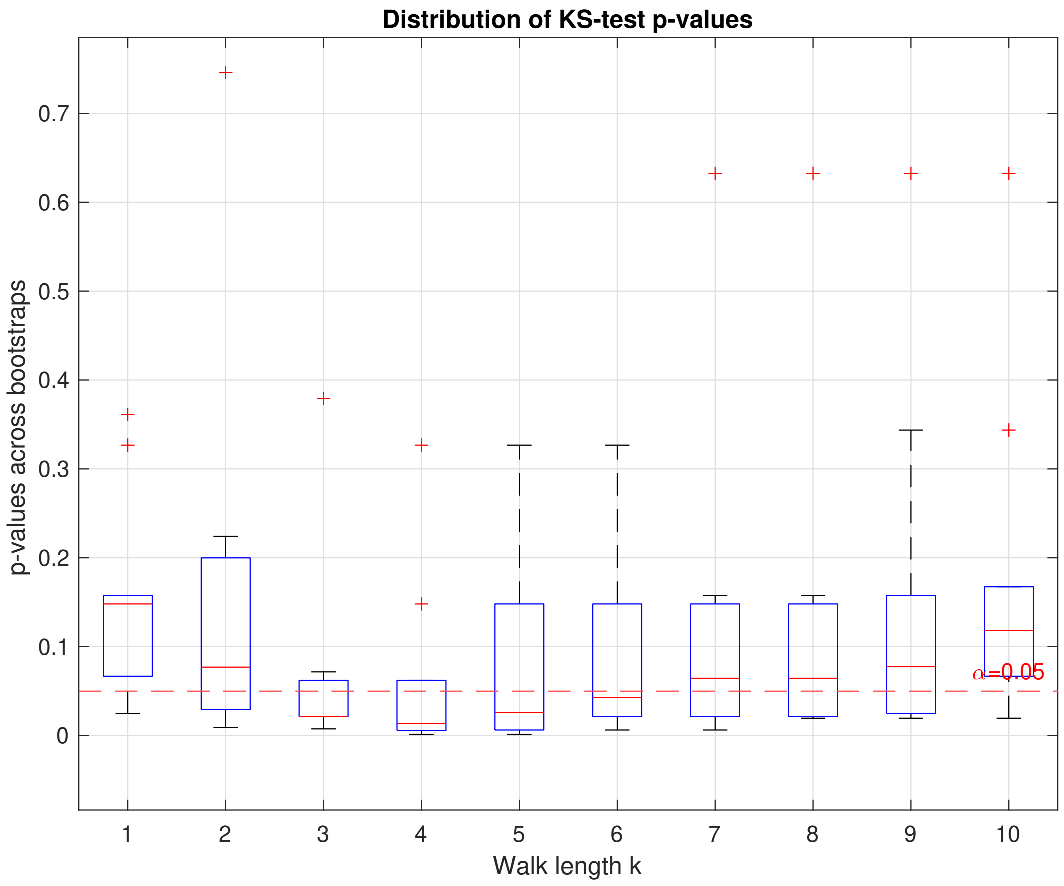

Figure 11. Distribution of two-sided Kolmogorov–Smirnov (KS) test p-values across bootstrap replicates for each walk length $k \in \{1, \ldots, 10\}$. For each subject's functional connectivity matrix, 10 surrogate datasets were generated using non-parametric bootstrapping of the BOLD time series, yielding multiple realizations of the original analysis.

8.1.2 One sided Bootstrap

To test for group differences assuming a specific direction, we repeated the bootstrap analysis using a one-sided KS test. This approach enables detection of divergence in the distributions of correlation values between migraine patients and controls, in a specific direction.

Figure 12 displays p-value distributions from both directional tests: Figure 12a tests whether patients exhibit stochastically lower correlations than controls, and Figure 12b tests the opposite.

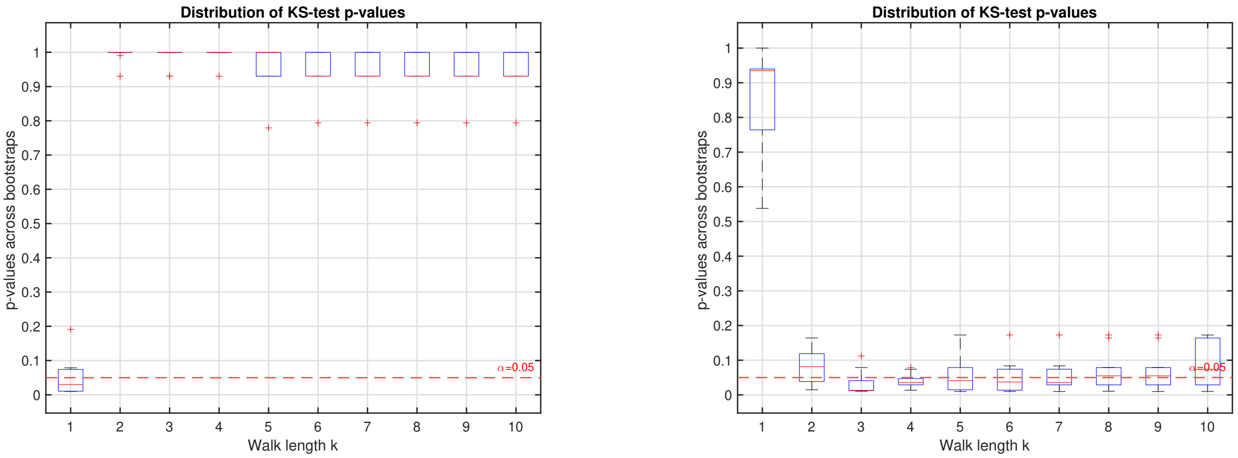

(a) KS test under the null hypothesis $H_0 : F_{\text{patient}}(x) \geq F_{\text{control}}(x)$. The alternative hypothesis is that the patient distribution is stochastically less.

(b) KS test under the null hypothesis $H_0 : F_{\text{patient}}(x) \leq F_{\text{control}}(x)$. The alternative hypothesis is that the patient distribution is stochastically greater.

Figure 12. Distributions of p-values from one-sided Kolmogorov–Smirnov (KS) tests across bootstrap replicates for each walk length k, under two complementary null hypotheses. In both panels, the dashed red line marks the $\alpha = 0.05$ significance threshold.

8.1.3 Block Bootstrap

We also applied a circular block bootstrap procedure to further validate our findings and account for temporal autocorrelation in the BOLD time series, a known limitation of standard bootstrapping methods. By resampling contiguous blocks of timepoints (block size = 5), this approach better preserves the short-range temporal dependencies while generating the new datasets.

For each subject, we created 10 block-bootstrap samples and recomputed their corresponding functional connectivity matrices F. These were then processed through the full structure-function mapping pipeline, and group-level statistics were re-evaluated across walk lengths $k = 1, \ldots, 10$.

The resulting distribution of p-values closely mirrors those obtained with conventional bootstrapping, confirming that the observed group differences, especially those at intermediate walk lengths, reflecting stable, reproducible effects.

9 Supplementary Tables

Anatomical Parcelation and Recording Sites

To create brain divisions, an atlas is used to establish the **ROIs**. These atlases provide standardized templates for segmenting the brain into distinct regions, which can then serve as nodes in the brain's connectome. Each **ROI** consists of **Voxels**, which are volumetric subdivisions of the brain, obtained through neuroimaging techniques.

Examples of an atlas are the **AAL**, which has several versions that focus on different brain regions[8–10],the Schaefer atlas[11], and the **Glasser-360** atlas[12].

For this study, will be used the Schaefer atlas[11], comprising 100 **ROIs**, supplemented by 30 additional regions from the **AAL116** atlas[8]. This inclusion ensures the coverage of key subcortical regions, which have been prominently associated with migraine studies[13]. Altogether, this results in a comprehensive set of 130 **ROIs**, detailed in table [1].

Nr	Region	Nr	Region	Nr	Region	Nr	Region
1	LH CCing 1	2	RH CCing 1	3	LH CPFLat 1	4	RH CPFLat 1
5	RH CPFLat 2	6	RH CPFLat 3	7	RH CPFLat 4	8	RH CPFMed 1
9	LH CPar 1	10	RH CPar 1	11	RH CPar 2	12	LH CPrec 1
13	RH CPrec 1	14	LH DPreFC 1	15	LH DPreFC 2	16	LH DPreFC 3
17	LH DPreFC 4	18	LH DPreFC 5	19	LH DPreFC 6	20	LH DPreFC 7
21	RH DPFDorM 1	22	RH DPFDorM 2	23	RH DPFDorM 3	24	RH DPFVent 1
25	RH DPFVent 2	26	LH DPar 1	27	RH DPar 1	28	LH DPar 2
29	LH DTemp 1	30	RH DTemp 1	31	LH DTemp 2	32	RH DTemp 2
33	RH DTemp 3	34	LH DPrecPC 1	35	RH DPrecPC 1	36	LH DPrecPC 2
37	RH DPrecPC 2	38	LH DAFEF 1	39	RH DAFEF 1	40	LH DAPost 1
41	RH DAPost 1	42	LH DAPost 2	43	RH DAPost 2	44	LH DAPost 3
45	RH DAPost 3	46	LH DAPost 4	47	RH DAPost 4	48	LH DAPost 5
49	RH DAPost 5	50	LH DAPost 6	51	LH DAPost 1	52	RH DAPost 1
53	LH LimbOF 1	54	RH LimbOF 1	55	LH LimbTP 1	56	RH LimbTP 1
57	LH LimbTP 2	58	LH SVAFI 1	59	RH SVAFI 1	60	LH SVAFI 2
61	LH SVAMed 1	62	RH SVAMed 1	63	LH SVAMed 2	64	RH SVAMed 2
65	LH SVAMed 3	66	LH SVAPF 1	67	LH SVAPO 1	68	RH SVATOP 1
69	RH SVATOP 2	70	LH SM 1	71	RH SM 1	72	LH SM 2
73	RH SM 2	74	LH SM 3	75	RH SM 3	76	LH SM 4
77	RH SM 4	78	LH SM 5	79	RH SM 5	80	LH SM 6
81	RH SM 6	82	RH SM 7	83	RH SM 8	84	LH Vis 1
85	RH Vis 1	86	LH Vis 2	87	RH Vis 2	88	LH Vis 3
89	RH Vis 3	90	LH Vis 4	91	RH Vis 4	92	LH Vis 5
93	RH Vis 5	94	LH Vis 6	95	RH Vis 6	96	LH Vis 7
97	RH Vis 7	98	LH Vis 8	99	RH Vis 8	100	LH Vis 9
101	LH Hippo 1	102	RH Hippo 1	103	LH Amyg 1	104	RH Amyg 1
105	LH Caud 1	106	RH Caud 1	107	LH Put 1	108	RH Put 1
109	LH Pall 1	110	RH Pall 1	111	LH Thal 1	112	RH Thal 1
113	LH Crus1 1	114	RH Crus1 1	115	LH Cer 3	116	RH Cer 3
117	LH Cer 4-5 1	118	RH Cer 4-5	119	LH Cer 6	120	RH Cer 6
121	LH Cer 6	122	RH Cer 6	123	Vermis 1-2	124	Vermis 3
125	Vermis 4-5	126	Vermis 6	127	Vermis 7	128	Vermis 8
129	Vermis 9	130	Vermis 10				

Table 1. Table of brain regions with corresponding numbers. This table lists numbered brain regions with their respective abbreviations, organized by left hemisphere (LH) and right hemisphere (RH) designations, as well as specific anatomical and functional regions.

9.1 NCT Results Tables

The following two tables provide the full numeric output from the CBIG Network Correspondence Toolbox (NCT)[6] analyses described in Section "Computing the spatial localization of network-level alterations" of the main article. For each of the four atlas/network-label combinations—AS400Y17, AS400K17, AS200K17 and AS200Y17—we list the Dice coefficient quantifying the spatial overlap between every Intrinsically Connectedness (IC) map and each of the 17 canonical functional networks, together with the associated spin-test p-value. An asterisk next to a p-value indicates statistical significance at $\alpha = 0.05$.

Table 2. Dice coefficients and p-values for AS400Y17 and AS400K17 conditions

AS400Y17			AS400K17		
Name	Dice	p-value	Name	Dice	p-value
TempPar	0.1251	0.048*	DefaultA	0.1745	0.1279
DefaultC	0.0867	0.1508	DefaultB	0.1531	0.0579
DefaultB	0.1727	0.0679	DefaultC	0.1316	0.0739
DefaultA	0.1600	0.2867	Language	0.0888	0.2138
ControlC	0.0663	0.049*	ControlA	0.0714	0.7223
ControlB	0.2296	0.007*	ControlB	0.1985	0.016*
ControlA	0.0271	0.9810	ControlC	0.0941	0.6124
LimbicA	0.1266	0.1768	Sal/VenAttnA	0.0809	0.4835
LimbicB	0.0202	0.8072	Sal/VenAttnB	0.1082	0.5844
Sal/VenAttnB	0.0843	0.5714	DorsAttnA	0.0902	0.6563
Sal/VenAttnA	0.0723	0.8202	DorsAttnB	0.1008	0.1249
DorsAttnB	0.1107	0.1688	Auditory	0.0669	0.4515
DorsAttnA	0.1042	0.4276	SomatomotorA	0.0625	0.4945
SomatomotorB	0.0632	0.5884	SomatomotorB	0.0693	0.2897
SomatomotorA	0.0924	0.4366	VisualA	0.0674	0.8312
VisualB	0.0385	0.7872	VisualB	0.0387	0.8442
VisualA	0.0618	0.8172	VisualC	0.0507	0.4406

Table 3. Dice coefficients and p-values for AS200K17 and AS200Y17 conditions

AS200K17			AS200Y17		
Name	Dice	p-value	Name	Dice	p-value
DefaultA	0.1660	0.1139	TempPar	0.1052	0.033*
DefaultB	0.1549	0.2707	DefaultC	0.0796	0.1598
DefaultC	0.1225	0.042*	DefaultB	0.1620	0.0829
Language	0.1063	0.1019	DefaultA	0.1369	0.2567
ControlA	0.0682	0.8911	ControlC	0.0657	0.0909
ControlB	0.2046	0.009*	ControlB	0.2724	0.001*
ControlC	0.0869	0.6823	ControlA	0.0525	0.9610
Sal/VenAttnA	0.0874	0.4615	LimbicA	0.1214	0.2557
Sal/VenAttnB	0.1023	0.5495	LimbicB	0.0183	0.8961
DorsAttnA	0.1024	0.5904	Sal/VenAttnB	0.0696	0.5904
DorsAttnB	0.0911	0.1089	Sal/VenAttnA	0.0807	0.8212
Auditory	0.0574	0.4835	DorsAttnB	0.1118	0.0679
SomatomotorA	0.0690	0.4416	DorsAttnA	0.1117	0.3836
SomatomotorB	0.0676	0.3357	SomatomotorB	0.0611	0.6374
VisualA	0.0618	0.7932	SomatomotorA	0.0951	0.4286
VisualB	0.0394	0.8092	VisualB	0.0419	0.7163
VisualC	0.0619	0.3417	VisualA	0.0587	0.8452

References

1. Seguin, C., Sporns, O. & Zalesky, A. Brain network communication: concepts, models, and applications. *Nat. Rev. Neurosci.* **22**, 625–639, DOI: 10.1038/s41583-021-00539-3 (2021).
2. Becker, C. O. *et al.* Spectral mapping of brain functional connectivity from diffusion imaging. *Sci. Reports* **8**, 1411, DOI: 10.1038/s41598-017-18769-x (2018).
3. Strang, G. *Linear Algebra and Its Applications* (Wellesley–Cambridge Press, Wellesley, MA, 2006), 4th edn.
4. Boumal, N., Mishra, B., Absil, P.-A. & Sepulchre, R. manopt, a MATLAB toolbox for optimization on manifolds. *arXiv preprint arXiv:1308.5200* (2013). 1308.5200.
5. Boyd, S. & Vandenberghe, L. *Convex Optimization* (Cambridge University Press, Cambridge, 2004).
6. Kong, R., Spreng, R. N., Xue, A. *et al.* A network correspondence toolbox for quantitative evaluation of novel neuroimaging results. *Nat. Commun.* **16**, 2930, DOI: 10.1038/s41467-025-58176-9 (2025).
7. Alexander Bloch, A. F. *et al.* On testing for spatial correspondence between maps of human brain structure and function. *NeuroImage* **178**, 540–551, DOI: 10.1016/j.neuroimage.2018.05.070 (2018).
8. Tzourio-Mazoyer, N. *et al.* Automated anatomical labeling of activations in spm using a macroscopic anatomical parcellation of the mni mri single-subject brain. *NeuroImage* **15**, 273–289, DOI: 10.1006/nimg.2001.0978 (2002).
9. Rolls, E. T., Joliot, M. & Tzourio-Mazoyer, N. Implementation of a new parcellation of the orbitofrontal cortex in the automated anatomical labeling atlas. *NeuroImage* **122**, 1–5, DOI: 10.1016/j.neuroimage.2015.07.075 (2015).
10. Rolls, E. T., Huang, C.-C., Lin, C.-P., Feng, J. & Joliot, M. Automated anatomical labelling atlas 3. *NeuroImage* **206**, 116189, DOI: 10.1016/j.neuroimage.2019.116189 (2020).
11. Schaefer, A. *et al.* Local-global parcellation of the human cerebral cortex from intrinsic functional connectivity MRI. *Cereb. Cortex* **28**, 3095–3114, DOI: 10.1093/cercor/bhx179 (2018).
12. Fan, L. *et al.* The human brainnetome atlas: A new brain atlas based on connectional architecture. *All Fac.* (2016).
13. Meylakh, N., Marciszewski, K., Pietro, F. K. D. *et al.* Deep in the brain: Changes in subcortical function immediately preceding a migraine attack. *Hum. Brain Mapp.* **39**, 2209–2219, DOI: 10.1002/hbm.24030 (2018).

Acknowledgements

We acknowledge the Portuguese Science Foundation and LARSyS for their support of our study. We are also thankful to the participants and to our collaborators from Hospital da Luz for their valuable contributions.

Author contributions statement

Gonçalo Grácio (Research design, Methodology development and implementation, Investigation, Visualization, Reference review, Writing – original draft); Sérgio Pequito and Rita Nunes (Investigation, Methodology development, Reference review, Software validation, Project administration); Patrícia Figueiredo (Funding acquisition, Project supervision, Data curation and preprocessing, Investigation, Reference review, Project administration); Ana Matoso and Inês Esteves (Data acquisition and preprocessing, Data curation, Investigation, Reference review); Ana R. Fouto and Amparo Ruiz-Tagle (Data acquisition and preprocessing); Raquel Gil-Gouveia (Funding acquisition, Supervision of data acquisition). All authors reviewed the manuscript.

Funding

Data acquisition work was supported by the Portuguese Science Foundation through grants 2023.03810.BDANA, SFRH/BD/139561/2018, COVID/BD/153268/2023, PTDC/EMD-EMD/29675/2017, and LISBOA-01-0145-FEDER-029675.

This work was also supported by LARSyS funding (10.54499/LA/P/0083/2020, 10.54499/UIDP/50009/2020, 10.54499/UIDB/50009/2020).

Additional information

Example data and the full analysis code supporting this study are available at: `https://github.com/ggoncalo02/Migraine-Paper`

Competing interests

The authors declare no competing interests.
